# The geometry of distributional preferences and a non-parametric identification approach: The Equality Equivalence Test^☆^

**DOI:** 10.1016/j.euroecorev.2015.01.008

**Published:** 2015-05

**Authors:** Rudolf Kerschbamer

**Affiliations:** Department of Economics, University of Innsbruck, Universitätsstrasse 15, A-6020 Innsbruck, Austria

**Keywords:** Distributional preferences, Social preferences, Other-regarding preferences, Social value orientations, Equality equivalence test

## Abstract

This paper proposes a geometric delineation of distributional preference types and a non-parametric approach for their identification in a two-person context. It starts with a small set of assumptions on preferences and shows that this set (i) naturally results in a taxonomy of distributional archetypes that nests all empirically relevant types considered in previous work; and (ii) gives rise to a clean experimental identification procedure – the *Equality Equivalence Test* – that discriminates between archetypes according to core features of preferences rather than properties of specific modeling variants. As a by-product the test yields a two-dimensional index of preference intensity.

“Everything should be made as simple as possible, but not one bit simpler” attributed to Albert Einstein (1879–1955).

## 1 Introduction

Many economists׳ default assumption is that all agents are exclusively motivated by their own material self-interest. This assumption is in sharp contrast to both day-to-day experience and empirical evidence gathered by psychologists and experimental economists in the last decades. This has aroused renewed interest in theories of other-regarding preferences, where arguments beyond material self-interest enter the decision maker׳s utility function.^1^ Typical examples of such arguments are other people׳s (material) well-being (as in distributional preferences models),^2^ others׳ opportunities and expected or observed behavior (as in reciprocity models),^3^ others׳ payoff expectations (as in guilt aversion models),^4^ or others׳ other-regarding concerns (as in type based models).^5^

The present paper focuses on the first of the above mentioned subclasses, i.e. on distributional (or “social”) preferences, where besides one׳s own material payoff the (material) well-being of others enters an agent׳s utility function. Distributional preferences have been shown to be behaviorally relevant in important market and non-market environments – see Sobel (2005) and Fehr and Schmidt (2006) for excellent surveys. The current paper adds to this literature by proposing (i) a *simple* classification of distributional preference types that nests almost all major classifications of archetypes discussed in the economic and the social psychology literature; and (ii) a *simple* identification procedure based on the classification.

Identification of distributional preferences has been the topic of numerous papers, of course – see Kerschbamer (2013) for a thorough review of the literature. These pioneering studies – which have greatly advanced our understanding of non-selfish behavior – suffer from at least one of two methodological shortcomings. First, the tests employed typically discriminate between the members of a somewhat arbitrary list of distributional types; and secondly, the identification procedures typically rely on strong structural assumptions.^6^

Regarding the former dimension – the *set of distributional types tested for* – previous studies either start with a given list of types, or they employ a test design that allows discriminating only between the members of a limited set of types.^7^ For instance, the path-breaking dictator-game study by Andreoni and Miller (2002) distinguishes between *selfish*, *Leontief* and *perfect substitutes* preferences, plus weak incarnations of those types; the follow-up study by Fisman et al. (2007) employs a richer design and discriminates between *self-interested*, *lexself* (lexicographic for self over other), *social welfare* and *competitive* types plus some mixes thereof; the pioneering discrete choice study by Engelmann and Strobel (2004) tries to disentangle *efficiency concerns* (defined as surplus maximization), *maximin* preferences and (two modeling variants of) *inequality aversion*; Blanco et al. (2011) discriminate between *selfish* and various intensities of piecewise linear *inequality aversion*; Charness and Rabin (2002), Cabrales et al. (2010) and Iriberri and Rey-Biel (2013) allow for *self-interested*, *social welfare*, *difference-averse* and *competitive* preferences; and the ring-test – originally developed by social psychologists to assess “social value orientations”^8^ and recently used by economists to identify type and intensity of distributional concerns^9^ – discriminates between *altruists*, *cooperators*, *individualists*, *competititors*, *aggressors*, *martyrs, masochists* and *sadomasochists.*

Turning to the second dimension – the *structural assumptions imposed* – the identification procedures employed in previous studies typically rely on strong assumptions regarding the form of the utility or motivational function meant to represent preferences. For instance, the ring-test is based on the assumption of *linear preferences*; the studies by Cabrales et al. (2010), Blanco et al. (2011) and Iriberri and Rey-Biel (2013) employ identification procedures based on the piecewise linear model originally introduced by Fehr and Schmidt (1999) as a description of self-centered inequality aversion and later extended by Charness and Rabin (2002) to allow for other forms of distributional concerns and thereby assume *piecewise linearity*; and Andreoni and Miller (2002), Fisman et al. (2007) and Cox and Sadiraj (2012) check consistency with – and estimate parameters of – standard or modified constant elasticity of substitution (*CES*) *utility functions*.

Summing up the above discussion we conclude (i) that there is neither an agreement in the literature on what the relevant set of distributional basic motivations – defined as the manner in which people care about the (material) well-being of others – is, nor on how to delimitate distributional types; and (ii) that existing studies employ identification procedures that rely on strong structural assumptions as, for instance, linearity, piecewise linearity or standard or modified CES forms. By using a systematic approach based on a small set of primitive assumptions on preferences, the present paper offers an improvement in both dimensions. It shows (i) that this set of assumptions naturally results in a well delineated, mutually exclusive and comprehensive distinction between nine archetypes of distributional concerns; and (ii) that this set gives rise to a simple non-parametric experimental test – the *Equality Equivalence Test* (*EET*) – that discriminates between the archetypes according to core features of preferences rather than properties of specific modeling variants or functional forms. As a byproduct the test yields a two-dimensional index of preference intensity.

While the primary purpose of this paper is methodological, the experimental results obtained in an implementation of the *EET* also produce some substantive insights. For instance, the result that – consistent with the theoretically appealing assumption that distributional preferences are convex – about 95% of the subjects reveal (weakly) more benevolent (less malevolent) preferences in the domain of advantageous than in the domain of disadvantageous inequality. A second interesting detail is that beyond selfish subjects, the empirically most frequent distributional archetypes are those who exhibit (at least weakly) positive attitudes towards others in both domains (i.e., altruism and maximin), while archetypes that imply a negative attitude in at least one of the domains are by far less important empirically (the behavior of less than a fourth of the subjects is consistent with any form of inequality aversion, for instance, and the choices of less than 7% of the subject population are consistent with spite).^10^

The rest of the paper is organized as follows: Section 2 presents the assumptions on which the analysis is based and argues that those assumptions are fulfilled by all major modeling variants of distributional preferences discussed in the economic and the social psychology literature. Section 3 introduces the proposed classification of preference types based on the rate an agent is willing to trade between own monetary payoff and the monetary payoff of another. Section 4 presents the proposed identification procedure – the “*Equality Equivalence Test*” (*EET*). It starts (in Section 4.1) by conveying the intuition behind the proposed identification approach and explaining its similarity to the Certainty Equivalence Test. Section 4.2 presents the symmetric basic version of the test, and Section 4.3 discusses several extensions. In Section 4.4 a two-dimensional index for identifying the archetype and characterizing the intensity of distributional concerns – the (*x*, *y*)*-*score – is introduced, and a graphical representation of the type–intensity distribution is proposed. Section 4.5 relates the (*x*, *y*)*-*score to other measures of type and intensity of distributional concerns. Section 5 illustrates the working of the *EET* by reporting experimental results generated with the symmetric basic version of the test, and Section 6 concludes. Implementation issues for the case where the test is used as a tool in experimental economics (to address research questions in which distributional preferences are expected to shape behavior, to control for subject pool effects, or to help to interpret data from other unrelated experiments) are discussed in Appendix A. Appendix B contains the instructions of the experiment reported in Section 5.

## 2 Assumptions

Let *a*=(*m*, *o*) denote an income allocation that gives material payoff *m* (for “my”) to the decision maker (DM or “agent”) and material payoff *o* (for “other”) to the other person. The space of feasible income allocations is assumed to be the non-negative orthant of *R*^2^ and is denoted by *A*. Throughout we assume that the DM is equipped with a preference relation over income allocations, which we denote by ≽. Technically, ≽ is a binary relation on *A*, allowing the DM to compare pairs of allocations *a*, a*∈*A*. We read *a*≽a* as “the DM weakly prefers allocation *a* to allocation a*” and denote the asymmetric and the symmetric part of ≽ by ≻ and ~, respectively.^11^ For the DM’s preferences we require:

Assumption 1 (completeness, transitivity and continuity): The DM’s preference relation on income allocations is complete, transitive and continuous. That is, for ≽ it holds that:

• for every pair *a*, *a*′∈*A*, either *a*≽*a*′*,* or *a*′≽*a* (or both);
• for every triple *a*, *a*′, a*∈*A*, if *a*≽*a*′ and *a*′≽a*, then *a*≽a*;
• for every two sequences *a*^1^, *a*^2^, *a*^3^,*…* and *a*′^1^, *a*′^2^, *a*′^3^,*…* in *A*, if the sequence *a*^1^, *a*^2^, *a*^3^,*…* converges to *a* and the sequence *a*′^1^, *a*′^2^, *a*′^3^,*…* converges to *a*′*,* and if *a*^*i*^≽*a*′^*i*^ for each *i,* then *a*≽*a*′.

Completeness (i.e., the first part of Assumption 1) requires that the DM can compare any two income allocations; transitivity (the second part) adds the requirement that the preferences of the DM are internally consistent; and continuity (the last part) says that the DM′s preferences do not exhibit “jumps”, with, for example, the DM preferring each element in the sequence *a*^1^, *a*^2^, *a*^3^,*…* to *a*′, but suddenly reversing her preferences at the limiting point of the sequence. While ordering (completeness and transitivity) is important for the arguments below (as it is for substantial parts of economic theory), continuity is not.^12^ As shown by Eilenberg (1941) the three parts of Assumption 1 together imply that the DM′s preferences can be summarized by means of a continuous utility or motivational function *u*(*m, o*) that assigns a real-valued index to every (*m*, *o*)∈*A*.

Assumption 2 (strict *m-*monotonicity): The DM′s preference relation on income allocations is strictly monotonic in the own material payoff. That is, comparing any two income allocations (*m*, *o*) and (*m′*, *o*) in *A* with the same level of *o*, (*m*, *o*)≻(*m*′, *o*)⇔*m>m*′ and (*m*, *o*)~(*m′, o*)⇔*m*=*m*′.

Strict *m-*monotonicity requires that – holding the material payoff of the other person constant – the DM strictly prefers more own material payoff to less own material payoff. This is quite a natural assumption. It is violated, for instance, if the DM is willing to burn her own monetary payoff because she feels bad whenever she has (much) more than the other person. Such behavior is essentially never observed in experiments. In terms of utility representation, Assumption 2 translates to the requirement that for every (*m*, *o*)∈*A* and ∆∈*R*_*++*_ we have *u*(*m*+*∆*, *o*)*>u(m*, *o*).

Assumption 3 (piecewise *o-*monotonicity): The DM′s preference relation between two income allocations that have the same own material payoff for the DM but different payoffs for the other person depends only on whether the DM is ahead or behind. That is, comparing any two income allocations (*m*, *o*) and (*m*, *o*′) in *A* with the same level of *m* and *o<o*′*,* the DM′s preference relationship between (*m*, *o*) and (*m*, *o*′) (i.e., whether ≻, ≺, or ~ holds) is constant for all *o*, *o*′, *m* such that *o>m* and is also constant for all *o*, *o*′ *m* such that *m*>*o*′ (but potentially different between the two domains).

Piecewise *o*-monotonicity requires that the DM′s general attitude towards the other person (i.e., whether she is benevolent, neutral, or malevolent to the other) depends only on whether the other person has more or less monetary payoff than the DM herself. In terms of utility representation, it translates to the requirement that for every ∆∈*R*_*++*_ the sign of the difference *u(m*, *o+*∆)*−u(m*, *o*) is constant for all (*m*, *o*)∈*A* with *o>m* and is also constant for all *(m*, *o*)∈*A* with *o+*∆*<m* (but potentially different between the two domains).

Piecewise *o*-monotonicity is both permissive and restrictive, depending on the perspective. It is permissive because it allows for all major variants of distributional preferences that have been discussed in the economic literature – see the discussion at the end of this and in the next section. Piecewise *o*-monotonicity is also restrictive because it implies (i) that preferences only depend on monetary outcomes, not on the way they are achieved (this is the defining feature of distributional preferences); and (ii) that the reference point for the evaluation of allocations (if one is used) is an equal-material-payoffs allocation.

Ad (i): The implication that preferences only depend on monetary outcomes is likely to be violated in many important applications. For instance, in strategic interactions (where the other person has an opportunity to move and thereby a possibility to influence the payoff of the DM) *beliefs about intentions behind observed or expected action choices* of the other person potentially play a role (see the literature on reciprocity and related concepts cited in Footnote 2). Also, in some games *beliefs about the payoff expectations of the other person* seem to influence behavior (see the literature on guilt aversion and related concepts cited in Footnote 3). Furthermore, in a richer environment, where agents have more information on each other, *beliefs about the other-regarding concerns of the other person* may play a role (as in the literature on type-based models cited in Footnote 4). Finally, *features of the situation* (such as context, entitlements, properties of the outcome generating process, etc.) *or the DM* (such as a code of conduct, or a preference for honesty) might shape behavior. Knowing that all those factors might be behaviorally relevant in a richer environment, it seems important that distributional preferences are identified in a non-strategic setting and a neutral frame to avoid confounds. This is not to say that distributional preferences are unimportant in richer environments, of course, but rather that they cannot be unambiguously identified there.

Ad (ii): Some distributional archetypes discussed in real life and in the literature (most importantly, inequality aversion and egalitarian motives; maximin, Rawlsian and Leontief preferences; and envy) are inevitably defined in terms of a “reference location”, where the DM׳s general attitude towards the other changes from preferring higher payoffs for the other to preferring lower payoffs. In theory, this reference location can be anything (an interval, a point, or whatsoever), and it can differ among individuals. In existing models of reference-dependent distributional concerns, the reference location is a point, and the point is the egalitarian one for all individuals (see, for instance, Bolton, 1991; Mui, 1995; Fehr and Schmidt, 1999; Bolton and Ockenfels, 2000; or Charness and Rabin, 2002). While Assumption 3 is more agnostic than existing models of reference-dependent distributional concerns, it is still restrictive.^13^ For instance, there might exist individuals who consider it fair to get 20% more than others but unfair to get 30% more. Assumption 3 does not allow for this. While it would be feasible, in principle, to generalize Assumption 3 (and the test relying on it) so as to allow for heterogeneous reference points, this would seriously impair simplicity and transparency: Ultimately the aim of the paper is to propose a classification of subjects in distributional preference types that is helpful in organizing experimental data. For that purpose we need some kind of clustering and not a different distributional type for each single individual. Stated differently, as *any* model the approach proposed here is by design an abstraction of reality, and hence is deliberately constructed so as to *not* explain some behavior, in return for parsimony.

While parsimony calls for a unique reference point, it does not suggest equality as the reference point. Equality is suggested by normative considerations and by empirical evidence. The normative basis of equality as a reference point is discussed in some detail in Konow (1993) and in the working paper version of this article (Kerschbamer, 2013). Regarding empirical evidence Andreoni and Bernheim (2009, p. 1607f) cite several studies showing that equal sharing is common in the context of joint ventures among business firms, partnerships among professionals, share tenancy in agriculture, and bequests to children. They also provide evidence indicating that equality is a frequent outcome of negotiation and conventional arbitration in the field. In lab-experiments the assumption that the egalitarian outcome is somehow focal among subjects who change their general attitude towards others at some point seems even more natural than in the field: Subjects enter the laboratory as equals, their roles are assigned randomly and they have absolutely no information about each other. It seems therefore quite plausible that those subjects who attribute special meaning to an allocation (again, nothing in Assumption 3 requires them to do so) do this to the egalitarian one. And there is indeed considerable support for this assumption in existing experimental data. For instance, one of the stylized facts in standard dictator games is precisely that a sizeable fraction of the subject population voluntarily cedes exactly half of the pie to the recipient, and that very few subjects cede more (Camerer, 1997). This result survives even in experiments where the action space is continuous and where the price for giving is quite high (see Andreoni and Miller, 2002, for instance). The frequency of equal divisions is even higher in ultimatum games, where expectations about the “reference point” of the recipient enter the picture (see Camerer, 2003). While all this evidence indicates that the egalitarian outcome has something special for a substantial fraction of subjects, it does not tell us anything about the exact fraction of subjects for whom this is the case.^14^ But this is exactly (one of) the question(s) the proposed test aims to address.

Assumption 4 (strict *equal-material-payoff-*monotonicity): The DM′s preference relation on income allocations is strictly monotonic in both payoffs along the ray *m*=*o*. That is, comparing any two income allocations (*m*, *o*) and (*m*′, *o*′) in *A* with *m=o* and *m*′*=o*′, (*m*, *o*)≻(*m*′, *o*′)⇔*m>m*′ and (*m*, *o*)~(*m*′, *o*′)⇔*m=m*′.

Strict *equal-material-payoff-*monotonicity requires that more preferred allocations are reached when the payoffs of both agents are increased along the 45° line. In terms of utility representation it translates to the requirement that for every *z*∈*R*_*+*_ and ∆∈*R*_*++*_ we have *u*(*z+*∆, *z+∆*)*>u*(*z*, *z*). In combination with strict *m-*monotonicity, strict *equal-material-payoff*-monotonicity essentially rules out extreme forms of spite by putting an upper bound on the malevolence of the DM along the ray *m*=*o*.

As is easily checked, almost all (modeling) variants of distributional preferences discussed in the economics literature satisfy Assumptions 1–4, notable exceptions being lexself preferences (discussed by Fisman et al., 2007) which – in a strict interpretation – violate the continuity part of Assumption 1, and maximin (or Rawlsian, or Leontief) preferences (discussed by Andreoni and Miller, 2002; Charness and Rabin, 2002; or Engelmann and Strobel, 2004, for instance) which – in their purest form (but not in the form typically discussed in the literature) – violate strict *m*-monotonicity.^15^

## 3 Classification of distributional preferences: delineation of types and their core features

**Fig. 1:**
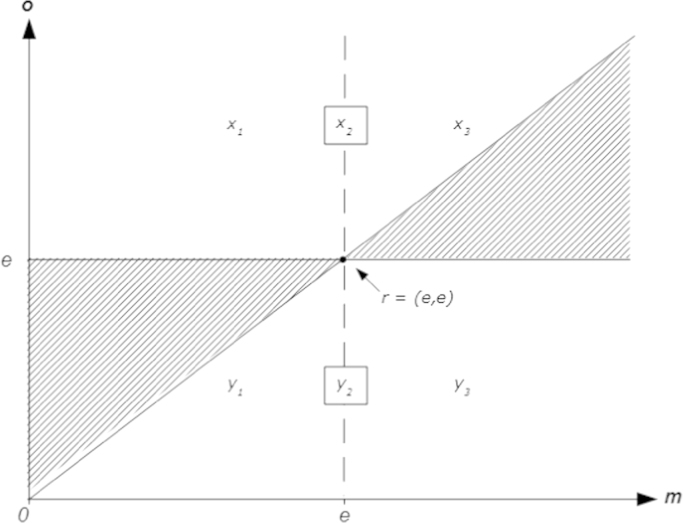
Delineation of archetypes of distributional preferences.

This section introduces a simple graphical classification of distributional preferences based on the four assumptions introduced in the previous section. Referring to Fig. 1, the preference of a DM is classified by characterizing the indifference curve that runs through the reference point *r*=(*e*, *e*). The choice space is divided into six relevant subsets, {*x*_1_, *x*_2_, *x*_3_} and {*y*_1_, *y*_*2*_*, y*_3_}. Here, *y*_1_ is the area below the 45° line and to the left of the vertical line through the reference point, *y*_2_ is the section of the vertical line that lies below the reference point, and *y*_3_ is the area to the right of the vertical line and below the horizontal line through the reference point. The subsets {*x*_1_, *x*_2_, *x*_3_} are defined similarly. Note that Assumptions 2 and 4 together imply that the indifference curve that runs through the reference point cannot pass through any of the two shaded areas in Fig. 1.

**Fig. 2:**
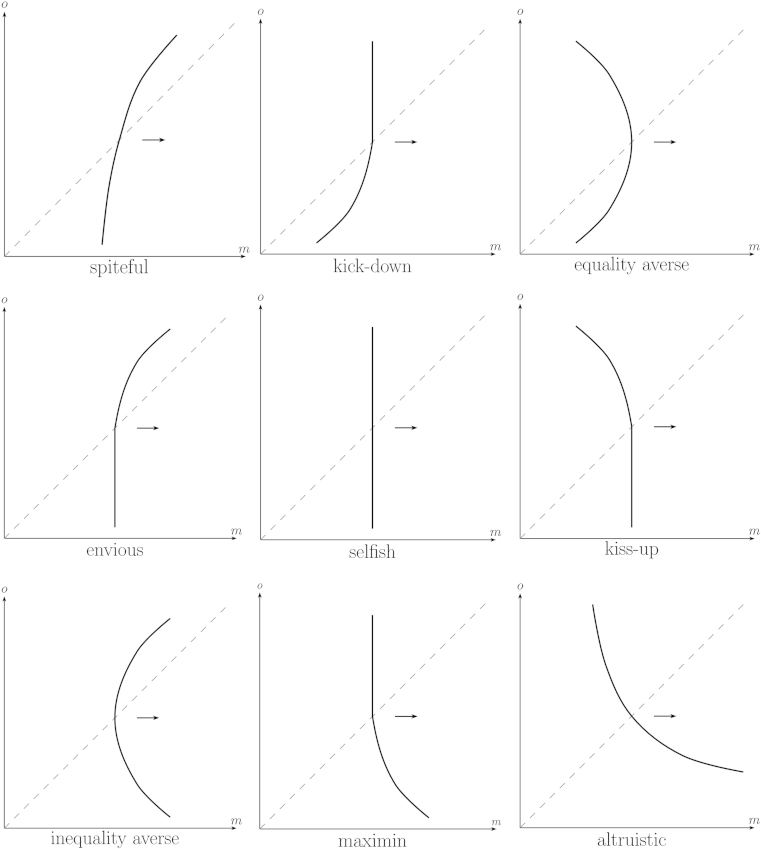
Typical indifference curves of the nine archetypes of distributional concerns. Arrows→indicate the locus of upper contour sets.

The preference type of the DM is now classified by the subsets that contain the DM’s indifference curve that runs through *r=*(*e*, *e*). Given Assumptions 1–4, one section of the indifference curve necessarily runs through one (and only one) of the *x* subsets, while the other section necessarily runs through one (and only one) of the *y* subsets. Therefore, it is simple to see that there are nine possible archetypes of distributional preferences given the proposed division in subsets. The nine archetypes are defined in Table 1 and a typical indifference curve of each archetype is displayed in Fig. 2. Let me shortly discuss the core features of different distributional preference types rattling around in the literature and how they fit into the proposed template.

• First consider *selfish* or *own-money-maximizing* preferences. They can be considered as a degenerated version of distributional preferences where an agent’s well-being neither increases nor decreases in the monetary payoffs of other agents. Thus, the core property of selfish preferences in a two-person context is that indifference curves in (*m*, *o*) space are vertical. Referring to Fig. 1 this means that a selfish DM’s indifference curve through *r*=(*e*, *e*) must run through the subsets *x*_2_ and *y*_2_ (as indicated in Table 1).
• The well-being of an *altruistic* agent increases in the monetary or utility payoffs of other agents (Becker, 1974; Andreoni and Miller, 2002); the well-being of an *efficiency loving* or *surplus maximizing* agent (Engelmann and Strobel, 2004), the well-being of an agent with *perfect substitutes* preferences (Andreoni and Miller, 2002) and the well-being of an agent with *social welfare* preferences (Charness and Rabin, 2002; Fisman et al., 2007) increases in the (weighted or unweighted) sum of payoffs. In all cases, well-being increases in *o* everywhere. Thus, indifference curves in (*m*, *o*) space are negatively sloped everywhere (if *o* increases *m* has to decrease to hold the agent indifferent) meaning that (in terms of Fig. 1) the indifference curve of an altruistic DM must pass through *x*_1_ and *y*_3_.
• An agent is *spiteful* (Levine, 1998), or *competitive* (Charness and Rabin, 2002), or *status seeking* or *interested in relative income* (Duesenberry, 1949), if her well-being decreases in the payoffs of others everywhere; so the core property of such preferences is positively sloped indifference curves in (*m*, *o*) space. In terms of Fig. 1 this means that a spiteful DM’s indifference curve through *r*=(*e*, *e*) must run through the subsets *x*_3_ and *y*_1_.
• The well-being of an *envious* or *grudging* agent decreases in the payoffs of agents who have more, but is unaffected by the payoffs of agents who have less (the role of envy has been emphasized by Bolton, 1991 and Mui, 1995, for instance); thus, the core property of envious preferences is positively sloped indifference curves in the domain of disadvantageous inequality and vertical indifference curves in the domain of advantageous inequality (yielding the combination *x*_3_, *y*_2_).^16^
• The well-being of an agent with *maximin* preferences (Engelmann and Strobel, 2004), *Rawlsian* preferences (Charness and Rabin, 2002), or *Leontief* preferences (Andreoni and Miller, 2002; Fisman et al., 2007) increases in the lowest of all agents’ payoffs. Thus, its defining feature in a two-person context is that indifference curves in (*m*, *o*) space are negatively sloped if inequality is advantageous and vertical otherwise (yielding the combination *x*_2_, *y*_3_).
• An agent is *inequity* or *inequality averse* (Fehr and Schmidt, 1999; Bolton and Ockenfels, 2000), or *difference averse* (Charness and Rabin, 2002; Fisman et al., 2007), or *egalitarian* (Dawes et al., 2007; Fehr et al., 2008) if she incurs a disutility when other agents have either higher or lower payoffs (as in the model by Fehr and Schmidt, 1999), or when the agent’s payoff differs from the average payoff of all agents (as in Bolton and Ockenfels, 2000). Consequently, the defining feature of inequality averse or egalitarian preferences in a two-person context is negatively sloped indifference curves in the domain of advantageous and positively sloped indifference curves in the domain of disadvantageous inequality (yielding the combination *x*_3_, *y*_3_).
• The opposite constellation, benevolence in the domain of disadvantageous inequality combined with malevolence in the domain of advantageous inequality, is referred to as *equality aversion* (by Hennig-Schmidt, 2002, for instance), or as *equity aversion* (e.g. by Charness and Rabin, 2002 and by Fershtman et al., 2012). Its defining feature in a two-person context is that indifference curves in (*m*, *o*) space are positively sloped below and negatively sloped above the 45° line (translating to *x*_1_, *y*_1_).

Table 1 lists and Fig. 2 displays two further archetypes of distributional preferences, “kick down” and “kiss up”. Those types have not been discussed in the literature and are included for completeness only:

• *Kick-down* or *bully-the-underlings* preferences imply malevolence towards agents who have lower and neutrality towards agents who have higher payoffs. Thus, the defining feature of such preferences in a two-person context is that indifference curves in (*m*, *o*) space are positively sloped in the domain of advantageous inequality and vertical in the domain of disadvantageous inequality (implying the combination *x*_2_, *y*_1_).^17^
• The opposite constellation, benevolence towards agents who are better off combined with neutrality towards those who are worse off, is called *kiss-up* or *crawl-to-the-bigwigs* preferences and such preferences imply negatively sloped indifference curves in the domain of disadvantageous inequality and vertical indifference curves in the domain of advantageous inequality (implying the combination *x*_1_, *y*_2_).

Note that the nine types listed in Table 1 and displayed in Fig. 2 are well delimitated, mutually exclusive and comprehensive. Also note how the four basic assumptions introduced earlier enter the picture: *ordering and continuity* translate into existence and uniqueness of indifference curves through any point in (*m*, *o*) space; *strict m-monotonicity* means that upper contour sets are to the right of an indifference curve (the arrows in Fig. 2); *piecewise o-monotonicity* requires that the general attitude of the DM (i.e., whether she is benevolent, neutral or malevolent) changes at most once – when crossing the equal-material-payoff line; and *strict equal-material-payoff-monotonicity* excludes indifference curves that fall on only one side of equal-material-payoff line. Thus, Assumptions 1–4 together naturally result in the distinction between the nine mutually exclusive and comprehensive archetypes listed in Table 1 and displayed in Fig. 2, meaning that qualitatively there is no room left for additional types.^18^

Before proceeding it seems important to address the potential critique that the nine archetypes defined here are not really new. This is correct, of course. The main contribution of the present paper is *not* to introduce new preference types; one of the goals is rather to derive the number and core properties of preference types from a small set of primitive assumptions on preferences. This stands in contrast to previous studies which either start with a given list of types or a specific model of preferences. A second – related – critique is that a list of archetypes similar to the one presented in Table 1 could also be obtained by working off the possible sign combinations of the two parameters in the piecewise linear model originally introduced by Fehr and Schmidt (1999) as a description of self-centered inequality aversion and later extended by Charness and Rabin (2002) to allow for other forms of distributional concerns. If one is willing to assume that subjects have preferences of this very specific form then this critique is justified. However, a major point in the current paper is exactly that there is no need to impose such a tight structure. This is true both for the type delineation introduced in this and the elicitation procedure proposed in the next section. Stated differently, all modeling variants of distributional preferences satisfying the four assumptions introduced in Section 2 and all distributional archetypes tested for in previous experiments fall into one of the nine categories defined here. This is also true for the Charness and Rabin model. On the other hand, there are many models of distributional preferences in the economic literature that do not fit into the piecewise linear framework of Charness and Rabin – the altruism models by Andreoni and Miller (2002), Cox et al. (2007) and Cox and Sadiraj (2012), the envy model by Bolton (1991), and the inequality aversion model by Bolton and Ockenfels (2000) are prominent examples.

## 4 Identification of distributional preferences: the *Equality Equivalence Test*

### 4.1 Idea of the Equality Equivalence Test

As mentioned earlier, the four basic assumptions introduced in Section 2 not only naturally result in a classification of distributional preference types that nests all major behavioral types discussed in the literature, but also give rise to a clean identification procedure (a “test”) that does not rely on unnecessary structural assumptions. This subsection explains how the test works and motivates its name (*Equality Equivalence Test*).

**Fig. 3:**
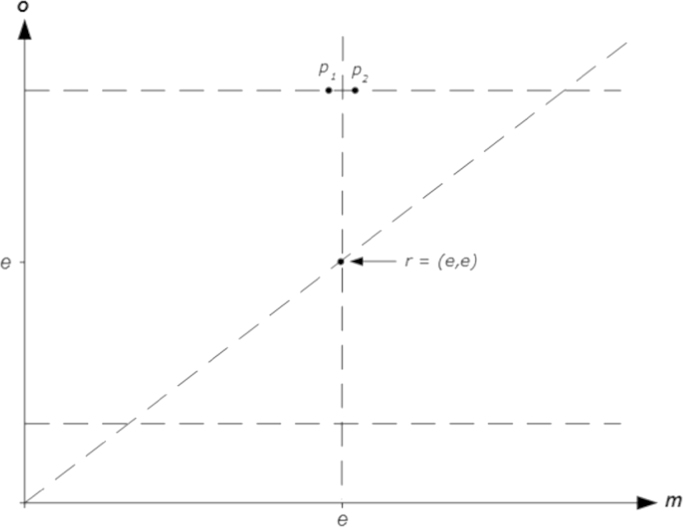
Identification of archetypes – the *Equality Equivalence Test*.

Given Assumptions 1–4, the DM’s type can be determined by identifying the location of the two sections of her indifference curve through the reference allocation *r*=(*e*, *e*), the section that passes the domain of disadvantageous inequality (the area above and to the left of the 45° line through the reference point) and the section that passes the domain of advantageous inequality (the area below and to the right of the 45° line). Theoretically, this can be done by exposing the DM to only four binary choices. Take points *r*, *p*_1_ and *p*_2_ in Fig. 3. Suppose we ask the DM to decide subsequently between *p*_1_ and *r* and between *p*_2_ and *r.* If the DM decides for the *p* allocation in both choices then she reveals *p*_1_≽*r* and *p*_2_≽*r*; thus, for the domain of disadvantageous inequality her indifference curve through *r*=(*e*, *e*) must run through *x*_1_. Similarly, if the DM reveals *r*≽*p*_1_ and *p*_2_≽*r* (by deciding for *r* in the first binary choice and for *p*_2_ in the second) then her indifference curve is in *x*_2_.^19^ And if the DM reveals *r*≽*p*_1_ and *r*≽*p*_2_ (by deciding for *r* in both choices) then her indifference curve is in *x*_3_.^20^ By exposing the DM in addition to binary choices between *r*=(*e*, *e*) and two points on the horizontal line below *r* (one to the left and one to the right of the vertical line through *r*) the location of the second part of her indifference curve through *r* – that is, the part that lies below the 45° line – can be determined. This is the idea behind the *EET*.

Note that the test proposed here is in many respects similar to the *Certainty Equivalence Test* (*CET*) used in experimental economics (and beyond) as a means to elicit risk attitudes (see Dohmen et al., 2010 for a recent application). With both procedures the DM is exposed to a short sequence of binary decision-making problems, where one of the two options is held constant across the binary choices. In the *CET* the recurring option is a coin-flip lottery (that is, a lottery with two possible outcomes occurring with the same probability) and the option that changes across choices is a safe amount of money. If the researcher is only interested in qualitative information about the risk attitude of a subject, then exposing her to just two binary choices – one in which the safe amount of money is just below the expected value of the lottery and another in which it is just above – is sufficient: If the subject decides for the lottery in both cases she is classified as risk-loving, if she decides for the lottery in the former choice and for the safe amount in the latter then she is classified as risk-neutral, and if she decides for the safe amount in both choices then she is classified as risk averse. This is very similar to the minimalist version of the *EET* described above, the main difference being that in the latter the attitude of the DM has to be elicited for two domains, for the domain of advantageous inequality and for the domain of disadvantageous inequality. An implication of this latter difference is that the minimum test size of the *EET* is four binary choices, while the minimum test size of *CET* is just two binary choices.

The minimal version of the *CET* (as described in the previous paragraph) gives only qualitative information about the risk attitude of the DM (it discriminates only between three types of DM – risk-averse, risk-neutral and risk-loving, where risk-neutrality cannot be identified exactly but only “with arbitrary precision”), just as the minimal version of the *EET* described previously gives only qualitative information about the distributional attitude of the DM (it discriminates only between the nine archetypes of distributional concerns listed in Table 1, where vertical parts of an indifference curve cannot be identified exactly but only “with arbitrary precision”).

The standard implementation of the *CET* differs from the minimal version described above in two respects: First it exposes subjects to more than one binary choice where the safe amount of money is higher (lower, respectively) than the expected value of the lottery; and secondly it includes one binary choice where the safe amount exactly equals the expected value of the lottery. The symmetric basic version of the *EET* (to be introduced in the next subsection) shares these two features: In terms of Fig. 3 (and focusing on the domain of disadvantageous inequality) it exposes subjects (i) to more than one choice between an option with the qualitative feature of *p*_1_ (*p*_2_, respectively); and (ii) to one choice where the alternative to the recurring reference point is located exactly on the intersection of the horizontal line above the reference point and the vertical line through the reference point. With both tests the aim of the former modifications (in comparison to the minimal version) is to get information about preference intensity while the latter modification is intended “to give a sign to neutrality” (see below).

The overall goal of the *CET* is to identify the safe amount that generates indifference to a given gamble. With a list of binary choices the point of indifference cannot be identified exactly. However, by keeping the lottery constant and increasing the safe amount systematically from one choice to the next the researcher can identify the “switching point” of the subject, i.e., the binary choice where the subject switches from the lottery to the safe alternative. This switching point gives a range for the point of indifference and thereby for the certainty equivalent of the subject to the given lottery. Suppose a subject decides for the lottery in all choices where the expected value of the lottery is higher than the safe amount and for the safe amount in all choices where it is lower. Then the behavior of the subject is consistent with risk neutrality. However, it is also consistent with a low degree of risk aversion and with a low degree of risk loving. By exposing the subject in addition to a choice where the safe amount corresponds to the expected value of the lottery the researcher “attaches a sign to risk neutrality”.

The overall goal of the *EET* is the identification of the locations of two points of indifference to the reference allocation, one for the domain of advantageous inequality, the other for the domain of disadvantageous inequality. With a list of binary choices the points of indifference cannot be identified exactly. However, by keeping the symmetric reference point and the material payoff of the other person in the asymmetric allocation constant across binary choices (in a given domain) and increasing the material payoff of the DM systematically from one choice to the next the researcher can identify the “switching point” of the subject in the respective domain, which gives a range for the point of indifference of the DM in the domain under scrutiny.^21^ As will be shown in Section 4.3, this information can be used to construct a two-dimensional index representing both the archetype of distributional concern and the preference intensity (conditional on the chosen vertical distance between *r* and the horizontal line). Suppose a subject decides for the symmetric reference point in all choices where her material payoff in the reference allocation is higher than her payoff in the asymmetric allocation and for the asymmetric allocation in all choices where it is lower. Then the behavior of the subject (in the domain under investigation) is consistent with selfishness. However, it is also consistent with a low degree of benevolence and with a low degree of malevolence. By exposing the subject in addition to a binary choice where her payoff is the same in the reference point and in the asymmetric allocation, we elicit her impartial distributional preference thereby “attaching a sign to selfishness”.^22^

Given the many similarities between *CET* and *EET* it probably does not come as a surprise that the two also share many pros and cons (in comparison to econometric elicitation techniques). The main advantages of the two tests are (i) that they are *simple* and *short* as they merely require subjects to complete a comparatively short sequence of binary decision making problems, properties that facilitate comprehension by experimental subjects and serve the experimenter’s need to limit the duration of experimental sessions; (ii) that they are *parsimonious* as they rely on a small set of comparatively mild primitive assumptions on preferences; (iii) that they are *general* as they directly tests the core features of preferences rather than concrete models or functional forms; (iv) that they are *flexible* as test size and test design can easily be fine-tuned to the research question of interest; (v) that they are *precise* because they identify the preference type with arbitrary precision and also give an index of preference intensity; and (vi) that they *minimize experimenter demand effects* as subjects are asked to make binary decisions in a neutral frame and do not have the option to do nothing. The main disadvantages of the two tests in comparison to econometric elicitation techniques are (i) that the switching point(s) of a subject give(s) only a range for the point(s) of indifference, which implies that “neutrality” cannot be identified exactly but only “with arbitrary precision”; (ii) that the assumptions on which the approaches rely are not directly tested; (iii) that the index of preference intensity for a given subject and the distribution of types that is inferred from a sample of subjects depend on the chosen parameterization of the test; and (iv) that they provide no measure of uncertainty of a subject’s elicited preference type. We discuss this latter issue further in Section 4.5.

### 4.2 The symmetric basic version of the *Equality Equivalence Test*

As explained above the *EET* exposes subjects to a series of diagnostic binary choice problems. In the (symmetric) basic version of the test the family of binary choices is characterized by four positive integers, *e*, *g*, *s* and *t*, where

**Fig. 4:**
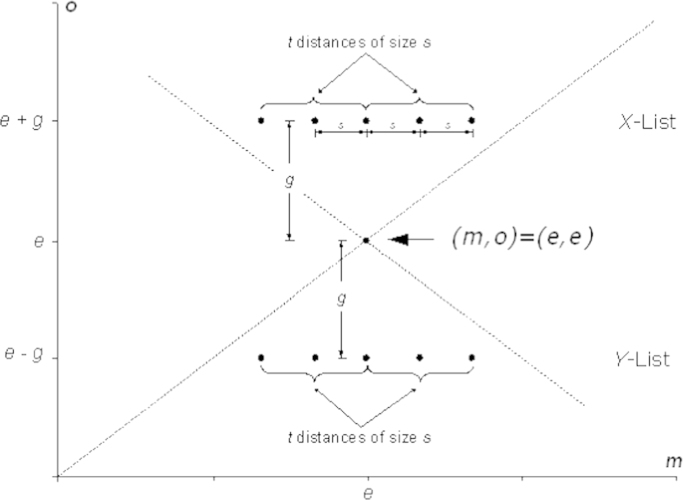
The geometry of the *Equality Equivalence Test*.

(i) *e* determines the locus of the *equal-material-payoff allocation* (*m*, *o*)=(*e*, *e*);
(ii) *g* is a “*gap*” *variable* characterizing the vertical distance between (*e*, *e*) and the two horizontal lines in Fig. 3 (see Fig. 4); in order to avoid zero or negative monetary payoffs we restrict *g* to values strictly smaller than *e;*
(iii) *s* is a “*step size*” *variable* characterizing the horizontal distance between two adjacent points on a line;
(iv) *t*≥1 is a “*test size*” *variable* determining the number of steps (of size *s*) which are made to the left and to the right starting from the point just above or below (*m*, *o*)=(*e*, *e*); in order to preserve advantageous and disadvantageous inequality we impose the restriction *t≤g*/*s.*

In total the symmetric basic version of the *EET* consists of 4*t*+2 binary decision problems. In each decision problem the subject is asked to decide between two alternatives (named Left and Right), each involving a payoff pair – one payoff for the subject (the DM) and one for the (randomly matched, anonymous) other subject (the passive person). For expositional purposes the decision problems are separated into two blocks, the disadvantageous inequality block (*X-*List) and the advantageous inequality block (*Y-*List). Within each block the decision problems are presented as rows in a table. In each decision problem one of the two alternatives (the alternative “Right”, say) is the (recurring) equal-material-payoff allocation (*m*, *o*)=(*e, e*). For the disadvantageous inequality block the second alternative in each decision problem (the alternative “Left”) is constructed as shown in Table 2. The construction of the second alternative for the advantageous inequality block is similar, the only difference being the material payoff of the passive person for the alternative LEFT, which is now *e−g* (instead of *e+g*).

An important feature of the *EET* is that within each of the two blocks the material payoff of the passive person in the asymmetric allocation is held constant, while the material payoff of the DM increases monotonically from one choice to the next. Together with the fact that the symmetric allocation remains the same in all choices, this design feature guarantees that strict *m*-monotonicity is enough to make sure that when facing the choice between Left and Right within a given block, each individual switches at most once from Right to Left (and never in the other direction). In Section 4.4 I will use the two switching points of a subject to construct a two-dimensional index representing both archetype and intensity of distributional concerns (conditional on the chosen test parameters – see Section 4.5 for a discussion).

As previously mentioned the *EET* allows for *discrimination between* the nine *archetypes at* any *arbitrary precision*. More specifically, the researcher needs to define when an agent should be considered as egoistic in a particular domain (this is the meaning of arbitrary precision). Suppose we define an agent to be egoistic in a particular domain if she is not willing to give up *c* Cents in order to change the material payoff of the passive person by 1$. Then the appropriate *EET* has to be such that *c=*100*s*/*g*⇔*s=cg*/100 meaning that we can choose the remaining parameters of the test freely.

### 4.3 Extending and refining the *Equality Equivalence Test*

The working paper version of this article (Kerschbamer, 2013) proposes three modifications of the symmetric basic version of the test that might help to shed light on more specific research questions. The first modification replaces the symmetric step-size in the basic version by an asymmetric one (where the step size is small at the center but grows larger when moving away from the center) in order to increase the power of the test to discriminate between selfish and different variants of non-selfish behavior without increasing the size of the test or decreasing the discriminatory power of the test at the borders. The second modification extends the *X*-List to the left and the *Y*-List to the right in order to address the question whether there are subjects who (in the relevant range) put more weight on the material payoff of the passive person than on their own material payoff. The third modification is a multi-list version of the *EET* where subjects are asked to complete two or more *X-* and *Y-*lists distinguished by the size of the gap variable *g.* This modification is intended to gain more insights on the exact shape of indifference curves in (*m*, *o*)-space.

### 4.4 Identifying archetype and characterizing intensity of distributional concerns: the (*x, y*)*-*score

This subsection describes a method to identify the archetype and to characterize the intensity of the distributional preference of a subject based on her choices in the symmetric basic version of the test. It then proposes a procedure to represent the type-intensity distribution of a given subject pool graphically.

**Fig. 5:**
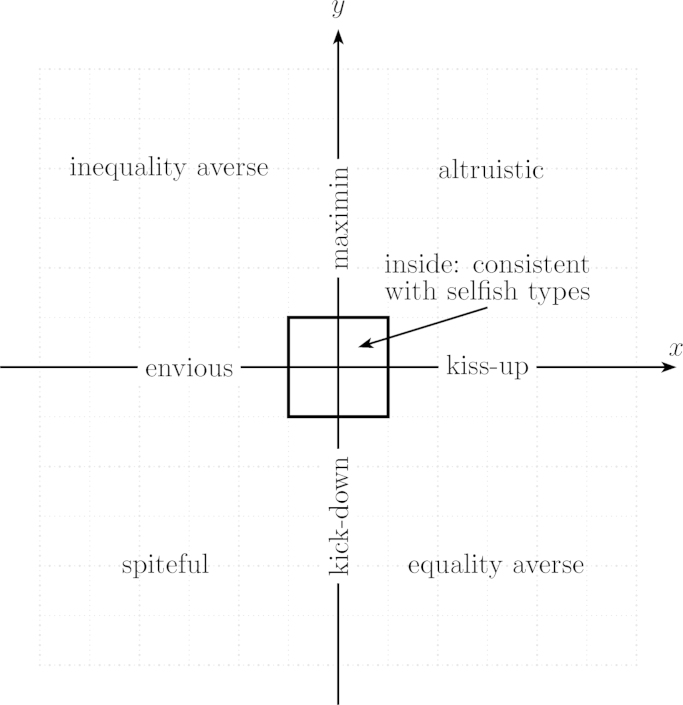
Distributional types in (*x*, *y*) space.

*Step 1* (*consistency check*): As argued above an individual whose preferences satisfy strict *m-*monotonicity has at most one switch from Right to Left (and no switch in the other direction) in each of the two tables. Step 1 is to eliminate all subjects that fail this basic consistency check (in an implementation of the symmetric basic version of the test – see Section 5 for details – less than 5% of the subjects failed the consistency check).
*Step 2* (*defining scores*): Represent each subject with consistent behavior by an (*x, y*) tuple defined as follows: The *variable x* (*x-score*) summarizes the behavior of the individual in the disadvantageous-inequality related block (*X-*List) and is defined as (*t*+1.5) points minus the row number in which the individual decides for the first time for the asymmetric allocation (that is, for the payoff vector on the left hand side). If an individual always decides for the symmetric (or egalitarian) allocation, we take the convention that she decides for the first time for the asymmetric allocation in the (2*t*+2)th row, so that she gets an *x*-score of −(*t*+0.5). For instance, if in the test version displayed in Fig. 4 (where *t*=2) an individual decides for the symmetric allocation in the first row of the *X*-List and for the asymmetric allocation in the second (and in all other) row(s) then she gets an *x-*score of 3.5–2=1.5. The *variable y* (*y-score*) summarizes the behavior of the subject in the advantageous-inequality related block (the *Y*-List) and is defined as the row number in which the individual decides for the first time for the asymmetric allocation minus (*t*+1.5) points. If an individual always decides for the symmetric allocation, we take again the convention that she decides for the first time for the asymmetric allocation in the (2*t*+2)th row; she then gets a *y*-score of *t*+0.5.
  Note that the definition of the two scores implies that each of them can take on 2(*t*+1) different values (see Table 3); thus, the proposed test allows for 4(*t*+1)² different (*x*, *y*)-scores. Also note that a positive (negative) *x-*score corresponds to benevolence (malevolence) in the domain of disadvantageous inequality, while a positive (negative) *y-*score corresponds to benevolence (malevolence) in the domain of advantageous inequality. Furthermore, the magnitude of the *x*-score (*y*-score, respectively) is an ordinal index of the intensity of distributional preferences in the domain of disadvantageous inequality (advantageous inequality, respectively).^23^
*Step 3* (*representing relative frequencies of types*): Represent the absolute or relative frequencies of the different (*x*, *y*)-scores in an axis of abscissas as shown in Fig. 5.

### 4.5 Relation of (*x*, *y*)-score to parameters in piecewise linear model and to WTP

The (*x, y*)-score as defined in the previous subsection is an ordinal index of preference intensity (a higher *x* means a higher weight on the other׳s payoff when the DM is behind while a higher *y* means a higher weight on the other׳s payoff when the DM is ahead) and as such is not normalized with respect to the four design parameters (*e*, *g*, *s*, *t*). This makes it difficult to compare the results of studies which use different sets of design parameters. This might be regarded as a drawback as the proposed test design is per se well suited for measuring the distributional preferences in experiments and representative surveys with large samples. To make the results of different studies comparable (even if they use different sets of design parameters) it might be advisable to replace the (*x*, *y*)-score by a cardinal metric that is equally easy to compute and has a similar intuitive interpretation. One way to get to such a metric is to translate the (*x*, *y*)-score into parameter ranges in structured models frequently used in the literature. The most widely used functional form in the empirical literature (see, for instance, Cabrales et al., 2010; Blanco et al., 2011; Iriberri and Rey-Biel, 2013) is the piecewise linear model introduced by Fehr and Schmidt (1999) as a description of self-centered inequality aversion and extended by Charness and Rabin (2002) to allow for other forms of distributional concerns. In the reciprocity free version the Charness and Rabin (CR) representation of preferences takes the form

$$u_{\gamma ,\sigma }(m,o)=(1-\sigma I_{m\leq o}-\gamma I_{m>o})m+(\sigma I_{m\leq o}+\gamma I_{m>o})o,\;\;\left(\mathrm{CR}\right)$$

where *γ* and *σ* are parameters assumed to satisfy *σ*≤*γ*<1 and where *I* is an indicator variable that takes the value of one if the condition in the subscript is met and the value of zero otherwise. This formulation says that the DM׳s utility is a linear combination of her own material payoff and the other person׳s material payoff and that the (otherwise constant) weight the DM puts on the other׳s payoff might depend on whether the other is ahead or behind. If one is willing to assume that subjects׳ preferences can be approximated by this form, how do (*x, y*)-scores in the symmetric basic version of the *EET* translate into parameter ranges in this model? This question is easily answered. Consider the *X*-List first. In this domain a DM with CR-preferences weakly prefers LEFT to RIGHT in row *r*∈{1,…, 2*t+*1} iff (1*−σ*)[*e+*(*r−t−*1)*s*]*+σ*(*e+g*)*≥e.* Thus, assuming that a DM who is indifferent decides for LEFT, the relationship between *x-*score and parameter range of *σ* in the piecewise linear model is as shown in Table 4. Using the same tie breaking rule (an indifferent DM decides for LEFT) for the *Y-*List we get a similar table (not shown) with *x-*score replaced by *y-*score, *σ* replaced by *γ*, and strict inequalities replaced by weak ones (and vice versa).

Note that the piecewise linear model implies that the DM’s willingness to pay (WTP) for income increases (or decreases) of the passive person is piecewise constant (WTP=*u*_*o*_/*u*_*m*_, where the subscripts denote partial derivatives). In the domain of disadvantageous (advantageous) inequality we have *WTP*^*d*^=*σ*/(1−*σ*) (*WTP*^*a*^=*γ*/(1−*γ*), respectively); if *σ*≥0 (*γ*≥0, respectively) then this term gives the own-money amount the DM is willing to give up in the domain of disadvantageous inequality (advantageous inequality, respectively) in order to *increase* the other person׳s material payoff by a single unit; symmetrically, if *σ*<0 (*γ*<0, respectively) then −*σ*/(1*−σ*) (−*γ*/(1−*γ*), respectively) gives the own-money amount the DM is willing to give up in the domain of disadvantageous inequality (advantageous inequality, respectively) in order to *decrease* the other person׳s material payoff by a single unit. Thus, within the piecewise linear model *x-*scores translate into *WTP*^*d*^ as shown in the right-most column of Table 4 (again the translation for the *y-*score is similar except that *WTP*^*d*^ is replaced by *WTP*^*a*^ and strict inequalities are replaced by weak ones).

It is important to note that using estimates of the parameters of the piecewise linear model (or estimates of the piecewise constant WTP for changes in the income of the other) as a cardinal metric for distributional preferences does not necessarily mean assuming piecewise linear preferences: In experimental set ups, where stakes tend to be small, the estimates are probably best interpreted as linear approximations of the true values. This interpretation is especially valid when the parameters of the piecewise linear model are estimated from the raw data using the McFadden (1974) random utility specification. In addition to yielding a cardinal metric that is comparable across studies, estimating the parameters of such a structural model has several other practical advantages as well:^24^

• As McFadden׳s random utility specification allows for noisy decisions, subjects with inconsistent choices do not have to be dropped. This may be crucial when a test design with high resolution (i.e. with large gap variable *g* and small step size *s*) is used, or when the binary choices are presented to the subjects one-at-a-time in random order (see Appendix A for a discussion of implementation issues).
• The parameters׳ standard errors enable statistical tests; for example, to check whether a subject׳s deviations from purely selfish behavior are statistically significant.
• The structural model could also be applied in the context of a finite mixture specification. This would allow the experimenter to identify the prevalent social preference types and to endogenously classify each subject into the type that fits her behavior best.
• The parameters of the piecewise linear model can also be estimated when the test is applied in its multi-list variant (introduced in Kerschbamer, 2013) where the (*x*, *y*)-score is no longer available.

## 5 Experimental results based on the symmetric basic version of the *EET*

Here the data from a paper-and-pen experiment based on the symmetric basic version of the test is reported. The experiment was conducted in paper-and-pen (and several other design features reported below were applied) to convince subjects that neither other experimental subjects nor the experimenters could identify the person who has made any particular decision. This was done in an attempt to minimize the impact of experimenter demand and audience effects. See List (2007) for a discussion on experimenter demand effects and Hoffman et al. (1994), Andreoni and Petrie (2004), and Andreoni and Bernheim (2009) for experimental evidence indicating that – depending on the experimental design – audience effects might have a large impact on subjects׳ behavior in dictator-game like situations.^25^

### 5.1 Experimental procedures

Five experimental sessions were conducted manually (i.e., in pen-and-paper) at the University of Innsbruck in autumn 2009. Forty subjects who had not participated in similar experiments in the past were invited to each session using the ORSEE recruiting system (Greiner, 2004). Since not all subjects showed up in time, 192 (instead of the invited 200) subjects from various academic backgrounds participated in total, and each subject participated in one session only. After arrival, subjects assembled in one of the two laboratories and individually drew cards with ID numbers (which remained unknown to other participants and the experimenters). Then instructions were distributed and read aloud.^26^ Instructions informed subjects (i) that there are two roles in the experiment, the role of an “active person” and the role of a “passive person”; (ii) that there is exactly the same number of active and passive subjects in the experiment and that roles are assigned randomly; (iii) that each active person is matched with exactly one passive person and vice versa, and that at no point in time a participant will get to know anything regarding the identity of the person she/he is matched with; (iv) that active persons are called to make a series of ten binary decisions that determine not only their own earnings from the experiment but also the earnings of the passive person they are matched with; (v) that passive persons do not have a decision to make in the experiment and that their earnings will depend exclusively on the decisions of the active person they are matched with; (vi) that only one of the ten choice problems of each active person will be relevant for cash payments; and (vii) that cash payments could be collected the day after the experiment at one of the secretaries who also handles the cash payments for other experiments (to ensure that the amount a subject earns cannot be linked to her/his decisions). Then subjects were randomly assigned to one of the two roles; active persons stayed in the same room while passive persons were escorted to the adjacent laboratory.

In both rooms subjects were seated at widely separated computer terminals (computers were switched off) with sliding walls. Active persons were handed out a form consisting of two pages – an empty cover sheet and a decision sheet as described in the next paragraph – and they were asked to fill out the decision sheet in private. Passive persons received a form consisting of three pages – an empty cover sheet and a two-page questionnaire unrelated to the experiment – and they were asked to complete the questionnaire in private. After the tasks in both rooms had been completed, for each active person one of the choice problems was randomly selected via a manual device – a bingo ball cage handled by the active person – for the purpose of cash-payment generation. The payoff-relevant decision problem was written on the cover page of the active person and the person was given the opportunity to take (in private) a look at her/his choice in the payoff-relevant decision problem. Now subjects in both rooms were asked to label (in private) the cover sheet of their document with their ID number. Then participants in both rooms were called to put their documents (again in private) in boxes before leaving the room. Anonymous cash payments started the next day – giving experimenters the opportunity to manually match active with passive persons in the meantime. Participants presented the card with their ID number to an admin staff person, who did not know who did what for which purpose nor how cash payments were generated, and they got their earnings in exchange (the fact that cash payments would be made that way was clearly indicated in the instructions). On average subjects earned approximately 11 Euros plus a show up fee of 4 Euros.

### 5.2 Experimental design

The symmetric basic version of the test was implemented with *e*=10, *g*=3, *s=*1*, t*=2 and with experimental currency units corresponding to Euros. Thus, each active person (96 in total) was exposed to 10 binary decision problems with (10, 10) as the recurring equal-material-payoff allocation. The decision problems were presented in two tables, 5 in the *X-*Table (disadvantageous inequality) and 5 in the *Y*-Table (advantageous inequality). The design of the two tables was similar to that of Table 2.

### 5.3 Experimental results

**Fig. 6:**
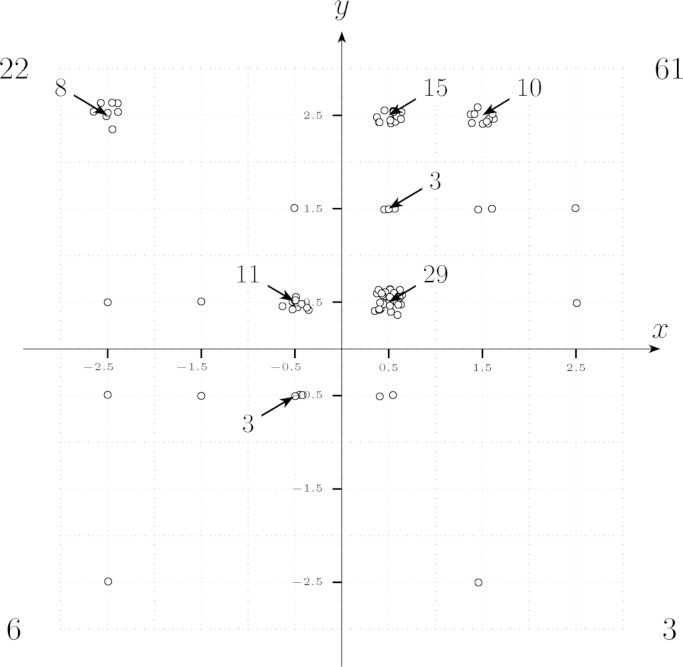
Absolute frequency of (*x*, *y*)-scores in experiments based on basic test version. (96 active persons; 4 revealed inconsistencies; the figure is based on the remaining 92 subjects.)

Of the 96 active subjects 4 (i.e., less than 5%) were eliminated in Step 1 of the procedure described in Section 4.3. The (*x*, *y*)-scores of the remaining 92 subjects were distributed as shown in Fig. 6.^27^ It is worth noting that more than half of the 36 points in the (*x*, *y*)-plane, where a subject could potentially sit, remain unoccupied, and only nine points are occupied by more than one subject. Thus, there is a sizeable amount of endogenous clustering. Also note that almost all subjects (87/92=95% of the population) reveal (weakly) more benevolent (less malevolent) preferences in the domain of advantageous than in the domain of disadvantageous inequality (i.e., their *y-*score weakly exceeds the *x-*score). Taken together those two pieces of evidence (endogenous clustering of subjects and decisions consistent with convex preferences) indicate that subjects understand the binary choices presented to them and that the results reported here are driven by well-behaved distributional preferences and not by noise. The second piece of evidence also implies that non-convex types (most importantly, kick down and equality averse) are empirically irrelevant. Turning to convex types (convexity refers to the shape of indifference curves here), it is interesting to note that the behavior of about two-thirds of the subjects (those in the positive quadrant; 61/92=66.30% of the subject population) is consistent with altruistic preferences (there are only 2 subjects who reveal non-convex altruism), while the behavior of (only) about one-fourth of the participants (those in the N/W quadrant; 22/92=23.92% of the subjects) is consistent with (any form of) inequality aversion.^28^ Spiteful subjects (negative quadrant) exist, but they account for less than 7% of our population (and even spiteful subjects׳ score is consistent with convex preferences).

It is also interesting to observe that the behavior of types at the border between altruism and inequality aversion (*x*∈{−$\frac{1}{2}$, $\frac{1}{2}$} and *y>*0; 59/92=64.13% of the subject population) is consistent with maximin, while the behavior of types at the border between inequality aversion and spite (*x<*0 and *y*∈{−$\frac{1}{2}$, $\frac{1}{2}$}; 18/92=19.57% of the population) is consistent with envy. Finally it is interesting to observe that the behavior of almost 50% of the population (those subjects with *x* and *y* in {−$\frac{1}{2}$, $\frac{1}{2}$}; 45/92=48.91% of the population) is consistent with selfish preferences. Here note that the test assigns selfish subjects to one of the four quadrants in Fig. 5 (Fig. 6, respectively) according to the “impartial distribution preference” expressed in the choice behavior in the (*t*+1)th row of the two lists (where the DM decides between two allocations that differ only in the payoff of the passive person – see Section 4.1 for a discussion). For instance, a subject that is weakly benevolent in both domains gets (*x*, *y*)=($\frac{1}{2}$, $\frac{1}{2}$), while a subject that is weakly benevolent when ahead but weakly malevolent when behind gets (*x, y*)=(−$\frac{1}{2}$, $\frac{1}{2}$). Looking at Fig. 6 we see that the choices of a majority of (but by far not the choices of all of) those subjects whose behavior is consistent with selfish preferences is also consistent with “lexself” as defined by Fisman et al. (2007).^29^

### 5.4 Discussion

The experiment reported here uses the fixed-role-assignment protocol, where roles (active DM and passive person) are assigned ex ante, and only active DMs decide while passive persons do nothing. While this protocol seems to be the cleanest one from a theoretical point of view, it is not practicable when the test is intended as a tool to be added to arbitrary other experiments, since the preferences of half of the subjects remain unclassified. An easy way to have a measure of all subjects׳ social preferences is to either use the role-uncertainty protocol (where each subject decides in the role of the active DM, and only later subjects get to know whether their decision is relevant – as in Engelmann and Strobel, 2004 and in Blanco et al., 2011, for instance), or the double-role-assignment protocol (where each subject decides, and each subject gets two payoffs, one as an active DM and one as a passive person – as in Andreoni and Miller, 2002 and in Fisman et al., 2007, for instance). Appendix A discusses some pros and cons of the different protocols. A second issue worth discussing regards the implemented test version. The experiment reported here uses the symmetric basic version of the test (which has equidistant step sizes in the binary choice lists) with a relative low resolution (i.e., a relatively high value of the quotient *s*/*g*). As is evident from the results, however, this form of the test yields a classification that is coarser than some researchers might find ideal. To address this issue, either an asymmetric test version with small step sizes in the center and larger step sizes in the periphery (as suggested in Section 4.3) could be used, or the power of the symmetric version of the test to discriminate between selfish and different variants of non-selfish behavior could be increased by increasing *g*, keeping the rest of the test as it is (remember the discussion on “identification with arbitrary precision” in Section 4.2). A third implementation issue regards the presentation of tasks. In the paper-and-pen experiments reported here the binary decision tasks were presented to the subjects in ordered lists (similar to the lists often used in risk-attitude elicitation tasks). In computer-aided experiments presenting the binary decisions one-at-a-time in random order (i.e., each binary decision on an own screen) might be an attractive alternative. Appendix A discusses this issue further. A final point worth addressing regards the comparison of subjects according to the intensity of preferences. The implemented standard version of the test uses only one size of *g.* If subjects differ in the shape of indifference curves, their relative ranking regarding preference intensity may depend on *g.* For instance, one person might be more altruistic than another if *g* is small, but less altruistic than the other if *g* is large. More generally, if distributional preferences are non-linear, any results regarding the relationship between intensity of distributional preferences and behavior in another experiment will depend to some degree on the level of *g* chosen for the test. So, if a researcher is interested in correlating the intensity of benevolence or malevolence in the two domains (i.e., the *x-* and the *y*-score) with behavior in another experiment it seems advisable to adapt the parameters of the test to the parameters in that other experiment.

## 6 Conclusions

This paper has proposed a geometric delineation of distributional preference types and a non-parametric approach for their identification in a two-person context. Major advantages of the proposed *Equality Equivalence Test* (*EET*) over previous ones are (i) that it is *simple* and *short* as subjects’ task is to make a small set of diagnostic choices without feedback; (ii) that it is *parsimonious* as it relies on a small set of primitive assumptions; (iii) that it is *general* as it directly tests the core features of different types of distributional preferences rather than concrete models or functional forms; (iv) that it is *flexible* as test size and test design can easily be fine-tuned to the research question of interest; (v) that it is *precise* as it identifies the archetypes of distributional concerns with arbitrary precision and also gives an index of preference intensity; and (vi) that it *minimizes experimenter demand effects* as subjects are asked to make binary decisions in a neutral frame and do not have the option to do nothing.^30^ Those features together suggest that the *EET* might be suitable as a tool in experimental economics to disentangle the impact of distributional preferences from that of other factors thereby helping to interpret data from other (unrelated) experiments (similar to the choice list tests used to elicit risk attitudes; see Holt and Laury, 2002, or Dohmen et al., 2010, 2011).^31^

That the *EET* is indeed suitable for that purpose has been shown in two recent studies: Balafoutas et al. (2012) investigate in a standard lab experiment the relationship between distributional preferences and competitive behavior and find (a) that distributional archetypes (as assigned by the proposed test) differ systematically – and in an intuitively plausible way – in their response to competitive pressure, in their performance in a competitive environment and in their willingness to compete; and (b) that controlling for the effects of distributional preferences, as well as for risk attitudes and some other factors, closes the large gender gap in competitive behavior found in earlier studies (by Niederle and Vesterlund, 2007, 2010, for instance). This is an important finding because it indicates that the gender gap in competitiveness is largely driven by mediating factors (potentially accessible to policy intervention) and not by gender per se. Hedegaard et al. (2011) examine in a large-scale internet experiment the impact of distributional concerns on the contribution behavior in a standard (linear) public goods game and find (a) that distributional archetypes differ systematically – and in an intuitively plausible way – in their contribution behavior; and (b) that accounting for the differences explains roughly half of the gap between actual behavior of subjects in the lab and the theoretical benchmark derived under the assumption that players are rational and selfish (and that this fact is common knowledge). Again, this is an important finding because it helps to disentangle the impact of distributional concerns on the behavior of subjects in social dilemma games from that of other factors – as beliefs on others׳ behavior or intentions, for instance. Together the findings in those studies clearly indicate that associating subjects with one of the proposed archetypes of distributional concerns has explanatory value and that the proposed test is indeed a valid control instrument in experimental economics.

Given that the *EET* does not provide a measure of uncertainty of a subject’s classification (in the sense of a counterpart of the standard error of an estimated parameter of a structural model), a systematic investigation of the test–retest reliability of the *EET* would be an interesting area of future research. The results of a recent study suggest that this reliability is high: Balafoutas et al. (2014) compare experimentally the revealed distributional preferences of individuals and teams by exposing subjects to the *EET* under two different decision-making regimes: an individual regime and a team regime. The authors employ a mixed within- and between-subjects design in two sets of sessions run in two consecutive weeks: In the first week all subjects are exposed to the *EET* in the individual regime; in the second week some subjects are again exposed to the individual regime, while the rest make their choices in the *EET* in the team regime. This design feature allows addressing the test–retest reliability issue by comparing the choices of subjects who face the individual regime twice across the two weeks. The authors show (in Table 5) that elicited preference types remain remarkably stable over the two weeks.

Beyond its potential to act as a control instrument in experimental economics, other potentially fruitful applications of the *EET* include (a) *investigating the stability of distributional preferences over different domains* (for instance, a potential shortcoming of the approach proposed here is its focus on the two-agents case; investigating whether the preferences revealed in that context carry over to a richer environment is surely an important issue);^32^ (b) *investigating possible links between distributional preferences and other forms of other-regarding preferences* (for instance, “Are altruists more or less likely to be motivated by positive or negative reciprocity?”, “Do altruism and altruistic rewarding (or altruistic punishment) go together or are they mutually exclusive ways to reach the same goal – promoting private provision of public goods?” 33, or “Is the test-based classification of subjects in distributional-preference types somehow correlated with the propensity to be motivated by trust?”);^34^ and (c) *applying the EET* (together with tests for risk and time preferences and for personality traits) *in experiments with large demographic variation* (age, gender, income, education) *or with a representative sample of the population to detect patterns and correlations* (for instance, “Are distributional preferences and risk attitudes or time preferences somehow related?”, “Are there gender differences in the distribution of archetypes?”^35^ or “What is the impact of age and income on distributional preferences?”). Beyond economics the proposed test might help to address important research questions in biology and psychology as, for instance, “What determines human altruism (or spite)?” or, “What drives altruistic punishments and rewards?”.

For those and many other interesting research questions, identification of distributional preference types in a “clean” environment appears to be a natural first step. The proposed *EET* seems to be well suited for this purpose. Turning back to the quote at the start of the paper the hope is that it turns out to be “*as simple as possible, but not one bit simpler*”.

## Figures and Tables

**Table 1:** Defining archetypes of distributional preferences.

**Preference type**	**Indifference curve passes**	
equality averse (equity averse)	*x*_1_	*y*_1_
kiss-up (crawl to the bigwigs)	*x*_1_	*y*_*2*_
altruistic (efficiency loving, surplus maximizing)	*x*_1_	*y*_*3*_
kick-down (bully the underlings)	*x*_2_	*y*_*1*_
selfish (own money maximizing)	*x*_2_	*y*_*2*_
maximin (Rawlsian, Leontief)	*x*_2_	*y*_*3*_
spiteful (competitive, status seeking, relative income m.)	*x*_3_	*y*_*1*_
envious (grudging)	*x*_*3*_	*y*_*2*_
inequality averse (inequity averse, egalitarian)	*x*_*3*_	*y*_*3*_

**Table 2:** The *X*-List (disadvantageous inequality).

Alternative: **Left**			Alternative: **Right**		
Please mark below if you prefer Left	You receive tokens	The passive person receives tokens	You receive tokens	The passive person receives tokens	Please mark below if you prefer Right
□	*e – ts*	*e*+*g*	*e*	*e*	□
…	*…*	*…*	*…*	*…*	…
□	*e – s*	*e*+*g*	*e*	*e*	□
□	*e*	*e*+*g*	*e*	*e*	□
□	*e*+*s*	*e*+*g*	*e*	*e*	□
…	*…*	*…*	*…*	*…*	…
□	*e*+*ts*	*e*+*g*	*e*	*e*	□

**Table 3:** Determination of (*x, y*)-score.

Subject chooses Left for the 1st time in row	In the *X*-list (*x*-score)	In the *Y*-list (*y*-score)
1	*t*+0.5	−(*t*+0.5)
2	*t*−0.5	−(*t*−0.5)
…	…	…
*t*	1.5	−1.5
*t*+1	0.5	−0.5
*t*+2	−0.5	0.5
…	…	…
2*t*+1	−(*t*−0.5)	*t*−0.5
Never	−(*t*+0.5)	*t*+0.5

**Table 4:** *x*-Score, parameter *σ* and willingness to pay (*WTP*^*d*^) in piecewise linear model.

***x-*****Score**		**Parameter range of*****σ*****in piecewise linear model**	***WTP***^***d***^**in piecewise linear model**
*t*+0.5	iff	*ts/*(*g+ts*) *≤σ*	*ts*/*g≤WPT*^*d*^
*t*−0.5	iff	(*t*−1)*s*/[*g+*(*t*−1)*s*] *≤σ<ts*/(*g+ts*)	(*t−*1)*s/g≤WPT*^*d*^*<ts/g*
…			
0.5	iff	0 *≤σ<s*/(*g+s*)	0 *≤WPT*^*d*^*<s/g*
−0.5	iff	−*s/*(*g*−*s*) *≤σ<* 0	−*s/g≤WPT*^*d*^*<* 0
…	…	…	…
−(*t*−0.5)	iff	−*ts/*(*g*−*ts*) *≤σ<* −(*t−*1)*s/*[*g*−(*t−*1)*s*]	−*ts/g≤WPT*^*d*^*<* −(*t−*1)*s*/*g*
−(*t*+0.5)	iff	*σ<* −*ts/*(*g*−*ts*)	*WPT*^*d*^*<* −*ts*/*g*

*t* is the test-size parameter in the *EET*; *s* is the step-size parameter in the *EET*; *g* is the gap-size parameter in the *EET*; *σ* is the weight the DM puts on the passive person׳s payoff in the domain of disadvantageous inequality in the piecewise linear model; *WTP*^*d*^: for *WPT*^*d*^> 0 this figure stands for the amount of own material payoff the DM is willing to give up in the domain of disadvantageous inequality in order to *increase* the other's material payoff by a unit; for *WPT*^*d*^<0 the absolute value of this figure stands for the amount of own material payoff the DM is willing to give up in the domain of disadvantageous inequality in order to *decrease* the other׳s material payoff by a unit (with inequalities reversed).

## Footnotes

1 While the prevailing assumption in the contemporaneous economic literature is that human behavior is exclusively motivated by material self-interest, in the early history of the profession it was not that uncommon to assume that “moral values” shape human behavior – see, e.g., Smith (1759) or Edgeworth (1881).

2 The major distributional (or social) preference types discussed in the literature are altruism (Becker. 1974; Andreoni and Miller, 2002) and surplus maximization (Engelmann and Strobel, 2004); inequality aversion (Fehr and Schmidt, 1999; Bolton and Ockenfels, 2000), difference aversion (Charness and Rabin, 2002) and egalitarian motives (Dawes et al., 2007; Fehr et al., 2008); maximin (Engelmann and Strobel, 2004), Rawlsian (Charness and Rabin, 2002) and Leontief preferences (Andreoni and Miller, 2002; Fisman et al., 2007); spiteful preferences (Levine, 1998) and concerns for relative income (Duesenberry, 1949); envy (Bolton, 1991; Kirchsteiger, 1994; Mui, 1995); and equity aversion (Charness and Rabin, 2002, Fershtman et al., 2012).

3 Positive (negative) reciprocity is the propensity to repay observed or expected generous (mean) actions of others by choosing actions that are generous (mean) to others. See Rabin (1993), Fehr et al. (1998), Charness and Rabin (2002), Dufwenberg and Kirchsteiger (2004), Falk and Fischbacher (2006), Bolle and Kritikos (2006), Cox et al. (2007, 2008) for theoretical models, and Fehr and Gächter (2000a) for an overview of experimental evidence and of implications of reciprocity.

4 In guilt aversion models people have a disposition to feel guilty when letting down others׳ payoff expectations implying that an agent׳s benevolence towards another person depends on what he believes that the other expects to receive (see Charness and Dufwenberg, 2006; Battigalli and Dufwenberg, 2007).

5 In type-based models the weight placed on the material payoff of another player depends on one׳s own (distributional) basic attitude as well as the perceived basic attitude of the other player. For instance, in Levine (1998) people are altruistic (or spiteful) to various degrees and – given their own basic altruism – they place more positive (negative) weight on the money received by an opponent who is believed to be more altruistic (spiteful). Andreoni and Bernheim (2009) have a similar model in which others׳ other-regarding concerns are evaluated in terms of inequality aversion (and not in terms of altruism).

6 One might argue that the list of distributional types tested for in the current paper is to some degree arbitrary, too. This is correct, of course. As will become clear below, the main innovation of the present paper in this regard is to derive the number and core properties of types from a small set of primitive assumption on preferences. This is in contrast to previous studies which either start with a given list of types or a specific model of preferences.

7 From the papers mentioned in the main text, Charness and Rabin (2002), Engelmann and Strobel (2004), Cabrales et al. (2010), Blanco et al. (2011) and Iriberri and Rey-Biel (2013) are examples of the former track (starting with a given set of types and designing tests to discriminate between the members of the set; or starting with a functional form and estimating the free parameters), while Andreoni and Miller (2002) and Fisman et al. (2007) are examples of the latter track (starting with a test without specifying a priori which types are tested for).

8 Standard references are Griesinger and Livingston (1973) and Liebrand (1984).

9 See, for instance, Offerman et al. (1996), Sonnemans et al. (1998), Van Dijk et al. (2002), Brosig (2002), Brandts et al. (2009), or Sutter et al. (2010).

10 The finding that very few subjects behave malevolently in variants of the dictator game is in line with previous studies – see Charness and Rabin (2002) or Engelmann and Strobel (2004), for instance.

11 As usual the asymmetric part of ≽ (“the DM strictly prefers allocation *a* to allocation *a**”) is defined by *a*≻*a**⇔*a*≽*a** but not *a**≽*a*; and the symmetric part (“the DM is indifferent between allocation *a* and allocation *a**”) is defined by *a*~*a**⇔*a*≽*a** and *a**≽*a.*

12 Continuity simplifies the presentation of the assumptions and the description of the core features of different archetypes of distributional preferences but is neither needed nor used in any other part of the paper. Continuity is not needed because the identification procedure proposed here uses information on the boundaries of revealed upper and lower contour sets to derive bounds on indifference sets. So, from a theoretical point of view, the procedure works even if indifference sets are singletons (as is the case for “lexself” preferences discussed by Fisman et al., 2007, for instance).

13 What Assumption 3 essentially requires is that the egalitarian outcome is somehow focal among those subjects who change their general attitude (i.e., whether they are benevolent, neutral or malevolent) towards others at some point. It does not require the attitude to change, though. In other words, while existing models of reference-dependent distributional concerns assume that “*something special happens at equality*”, Assumption 3 “only” requires that “*if something special happens with preferences then it happens around equality”.*

14 Here note that an egalitarian subject – according to the definition given in the next subsection – does not necessarily decide for an egalitarian allocation in a dictator game: If her preferences are smooth and satisfy strict *m*-monotonicity she will rather accept some advantageous inequality as this increases the utility derived from the own money component at a low cost in terms of the second component; similar arguments hold for other reference-dependent motives and other game forms.

15 The ERC model by Bolton and Ockenfels (2000) permits even violations of weak *m-*monotonicity. The same is true for (models of) some “social value orientation types” (the synonym for distributional preference types used by social psychologists), most notably, “martyrdom”, “masochism” and “sadomasochism”. It is important to note, however, that even in the social psychology literature violations of *m*-monotonicity are empirically irrelevant (I know of no study finding more than 5% of subjects in the mentioned categories).

16 Envy has also been discussed by Kirchsteiger (1994). His definition of envy corresponds to my definition of “spite”, though.

17 A basic disposition related to our “kick-down” preferences has recently been discussed under the heading “last-place aversion” (by Kuziemko et al., 2014, for instance). A last-place averse individual has a psychological disgust against being “last”, which creates a propensity for low-income individuals to punish individuals slightly below or above themselves, in the hope of keeping at least one agent below them.

18 There is room left for discussions on names, of course – see Footnote 16 for an example. And there is room left for discrimination within a given class; for instance, it might be interesting and important to discriminate between altruism and efficiency (or cake-size) concerns (both imply negatively sloped indifference curves in both domains), or between the Fehr and Schmidt (1999) and the Bolton and Ockenfels (2000) model of inequality aversion (both imply positively sloped indifference curves above and negatively sloped indifference curves below the 45° line). Although discrimination within a given class is not the main aim of the test proposed in the next section, the working paper version of this article (Kerschbamer, 2013) discusses a test version that might turn out to be helpful for this task as well.

19 Here note that the proposed elicitation procedure cannot identify a vertical section of an indifference curve exactly but only with “arbitrary precision” (more on this below).

20 Note that *strict m-monotonicity* implies *p*_2_≻*p*_1_. Thus, if a DM with preferences in line with Assumptions 1–4 decides for *p*_1_ in the binary choice between *p*_1_ and *r* then she will decide for *p*_2_ in the choice between *p*_2_ and *r.*

21 A DM who is very benevolent in the domain of advantageous (disadvantageous) inequality will decide for the *r* (the *p*) allocation in each binary choice between *r* and a point on the horizontal line below (above) *r.* Although such a DM will not switch in the respective domain her behavior still pins down the distributional type and gives a measure of the intensity of preferences.

22 Referring back to Fig. 3, if a DM reveals *r*≽*p*_1_ and *p*_2_≽*r* (by deciding for *r* in the former binary choice and for *p*_2_ in the latter) then her choices are consistent with selfishness in the domain of disadvantageous inequality. By exposing the DM in addition to a choice between the point located exactly halfway between *p*_1_ and *p*_2_ and the reference point we are implicitly asking her about her distributional attitude when no own money is involved. If she decides for the asymmetric (the symmetric) allocation then we can infer that she is weakly benevolent (malevolent) in the domain of disadvantageous inequality.

23 Also note that a score of +0.5 indicates weak benevolence, while a score of −0.5 indicates weak malevolence.

24 I thank an anonymous colleague for suggesting this interesting discussion.

25 See Hoffman et al. (1994) and Cox and Sadiraj (2012) for (almost double blind) experimental designs similar to the one employed here. Appendix A discusses alternative designs more suitable for the case where the *EET* is used as a tool in computer-supported experiments to address research questions in which distributional preferences play an important role, to control for subject pool effects, or to help to interpret data from other (unrelated) experiments. Those alternative protocols might be regarded as more problematic in terms of experimenter demand and audience effects but have advantages in other dimensions (an important one being that subjects are motivated to think about their decisions carefully and make choices that reflect their true preferences even when the preference elicitation procedure is only one of several tasks in an experiment).

26 The instructions are in Appendix B.

27 It is important to note that the data points in Fig. 6 are jittered (to make each single point visible). For instance, the 29 observations scattered around the point ($\frac{1}{2}$, $\frac{1}{2}$) all belong to the point ($\frac{1}{2}$, $\frac{1}{2}$).

28 Given that differences in the distribution of types between subject pools are likely to be large, this piece of evidence should not be interpreted as indicating that egalitarian motives are important only for a minority of subjects. See Fehr et al. (2006) for experimental evidence indicating that students (especially students of economics) are less egalitarian and more efficiency oriented than the rest of the population. See also the response by Engelmann and Strobel (2006) in the same issue.

29 Here note that in the *EET* a subject with lexself preferences necessarily ends up with an (*x*, *y*)-score of ($\frac{1}{2}$, $\frac{1}{2}$), independently of the parameterization of the test. As can be seen in Fig. 6 about one-third of the participants (specifically, 29 of the 92 classified subjects) ended up with such a score.

30 This is in contrast to the standard dictator game which gives the DM a windfall gain and then the option to share. This makes it pretty clear what would be considered decent behavior by the experimenter.

31 Some implementation issues relevant for this purpose are discussed in Appendix A.

32 As discussed in Section 4.1 the *EET* is in many respects similar to the standard risk-attitude elicitation procedures that ask subjects to compare a binary lottery either to another binary lottery (as in Holt and Laury, 2002) or to a risk-free alternative (as in Dohmen et al., 2010, 2011). In both domains, the risk-preference domain and the distributional-preference domain, the hope is that the preference type revealed in the binary environment is informative of the attitude of the decision maker in richer environments.

33 Altruistic rewarding (punishment) is the propensity to give rewards to (impose sanctions on) others for “norm-abiding behavior” (“norm-violating behavior”) even if rewards (sanctions) are costly for the rewarder (punisher) and yield no private material benefits whatsoever. See Fehr and Gächter (2000b, 2002), or Fehr and Fischbacher (2004), for studies investigating the power of altruistic rewards and punishments.

34 At first blush this avenue of research seems to be closely related to that of Blanco et al. (2011). It is not that close, however, since the research question proposed here is not whether the behavior of players in strategic games can be explained by distributional preferences (alone) but rather whether there is a link between distributional preferences and other forms of other-regarding preferences.

35 This research question is not new, of course – see Andreoni and Vesterlund (2001), for instance. The proposed approach allows for a more detailed investigation of this issue, though. And it is suitable to be integrated in large-scale experiments (as shown by Hedegaard et al. 2011).

## References

[bib1] Andreoni J., Bernheim D. (2009). Social image and the 50–50 norm: a theoretical and experimental analysis of audience effects. Econometrica.

[bib2] Andreoni J., Miller J. (2002). Giving according to GARP: an experimental test of the consistency of preferences for altruism. Econometrica.

[bib3] Andreoni J., Petrie R. (2004). Public goods experiments without confidentiality: a glimpse into fund-raising. J. Public Econ..

[bib4] Andreoni J., Vesterlund L. (2001). Which is the fair sex? Gender differences in altruism. Q. J. Econ..

[bib5] Balafoutas L., Kerschbamer R., Sutter M. (2012). Distributional preferences and competitive behavior. J. Econ. Behav. Organ..

[bib6] Balafoutas L., Kerschbamer R., Kocher M., Sutter M. (2014). Revealed distributional preferences: individuals vs. teams. J. Econ. Behav. Organ.

[bib7] Battigalli P., Dufwenberg M. (2007). Guilt in games. Am. Econ. Rev. Pap. Proc..

[bib8] Becker G. (1974). A theory of social interactions. J. Polit. Econ..

[bib10] Blanco M., Engelmann D., Normann H. (2011). A within-subject analysis of other-regarding preferences. Games Econ. Behav..

[bib11] Bolle F., Kritikos A. (2006). Reciprocity, altruism, solidarity: a dynamic model. Theory Decis..

[bib12] Bolton G. (1991). A comparative model of bargaining: theory and evidence. Am. Econ. Rev..

[bib13] Bolton G., Ockenfels A. (2000). ERC: a theory of equity, reciprocity, and competition. Am. Econ. Rev..

[bib14] Brandts J., Riedl A., van Winden F. (2009). Competitive rivalry, social disposition, and subjective well-being: an experiment. J. Public Econ..

[bib15] Brosig J. (2002). Identifying cooperative behavior: some experimental results in a prisoner’s dilemma game. J. Econ. Behav. Organ..

[bib17] Cabrales A., Miniaci R., Piovesan M., Ponti G. (2010). Social preferences and strategic uncertainty: an experiment on markets and contracts. Am. Econ. Rev..

[bib18] Camerer C. (1997). Progress in behavioral game theory. J. Econ. Perspect..

[bib19] Camerer C. (2003). Behavioral Game Theory: Experiments in Strategic Interaction.

[bib20] Charness G., Dufwenberg M. (2006). Promises and partnership. Econometrica.

[bib21] Charness G., Rabin M. (2002). Understanding social preferences with simple tests. Q. J. Econ..

[bib22] Cox J., Friedman D., Gjerstad S. (2007). A tractable model of reciprocity and fairness. Games Econ. Behav..

[bib23] Cox J., Friedman D., Sadiraj V. (2008). Revealed altruism. Econometrica.

[bib24] Cox J., Sadiraj V. (2012). Direct tests of individual preferences for efficiency and equity. Econ. Inq..

[bib26] Dawes Ch., Fowler J., Johnson T., McElreath R., Smirnov O. (2007). Egalitarian motives in humans. Nature.

[bib27] Dohmen Th., Falk A., Huffman D., Sunde U. (2010). Are risk aversion and impatience related to cognitive ability?. Am. Econ. Rev..

[bib28] Dohmen Th., Falk A., Huffman D., Sunde U., Schupp J., Wagner G. (2011). Individual risk attitudes: measurement, determinants and behavioral consequences. J. Eur. Econ. Assoc..

[bib30] Dufwenberg M., Kirchsteiger G. (2004). A theory of sequential reciprocity. Games Econ. Behav..

[bib31] Duesenberry J. (1949). Income, Saving and the Theory of Consumer Behavior.

[bib32] Edgeworth F. (1881). Mathematical Psychics: An Essay on the Application of Mathematics to the Moral Sciences.

[bib33] Eilenberg S. (1941). Ordered topological spaces. Am. J. Math..

[bib34] Engelmann D., Strobel M. (2004). Inequality aversion, efficiency, and maximin preferences in simple distribution experiments. Am. Econ. Rev..

[bib35] Engelmann D., Strobel M. (2006). Inequality aversion, efficiency, and maximin preferences in simple distribution experiments: reply. Am. Econ. Rev..

[bib36] Falk A., Fischbacher U. (2006). A theory of reciprocity. Games Econ. Behav..

[bib37] Fehr E., Bernhard H., Rockenbach B. (2008). Egalitarism in young children. Nature.

[bib39] Fehr E., Fischbacher U. (2004). Third-party punishment and social norms. Evol. Hum. Behav..

[bib40] Fehr E., Gächter S. (2000). Fairness and retaliation: the economics of reciprocity. J. Econ. Perspect..

[bib41] Fehr E., Gächter S. (2000). Cooperation and punishment in public goods experiments. Am. Econ. Rev..

[bib42] Fehr E., Gächter S. (2002). Altruistic punishment in humans. Nature.

[bib43] Fehr E., Kirchsteiger G., Riedl A. (1998). Gift exchange and reciprocity in competitive experimental markets. Eur. Econ. Rev..

[bib44] Fehr E., Schmidt K. (1999). A theory of fairness, competition, and cooperation. Q. J. Econ..

[bib45] Fehr E., Schmidt K., Kolm S., Ythier J.-M. (2006). The economics of fairness, reciprocity and altruism: experimental evidence.

[bib46] Fehr E., Naef M., Schmidt K. (2006). Inequality aversion, efficiency, and maximin preferences in simple distribution experiments: comment. Am. Econ. Rev..

[bib47] Fershtman Ch., Gneezy U., List J. (2012). Equity aversion: social norms and the desire to be ahead. Am. Econ. J.: Microecon..

[bib49] Fisman R., Kariv S., Markovits D. (2007). Individual preferences for giving. Am. Econ. Rev..

[bib50] Greiner B. (2004). The Online Recruiting System ORSEE 2.0 – A Guide for the Organization of Experiments in Economics. WP Series in Economics 10.

[bib51] Griesinger D., Livingston J. (1973). Toward a model of interpersonal motivation in experimental games. Behav. Sci..

[bib52] Hedegaard, M., Kerschbamer, R., Tyran, J.-R., 2011. Correlates and Consequences of Distributional Preferences: An Internet Experiment. Mimeo. Department of Economics, University of Copenhagen.

[bib53] Hennig-Schmidt H., Andersson F., Holm H. (2002). The impact of fairness on decision making - an analysis of different video experiments. Experimental Economics: Financial Markets, Auctions, and Decision Making.

[bib54] Hoffman E., McCabe K., Shachat K., Smoth V. (1994). Preferences, property rights, and anonymity in bargaining games. Games Econ. Behav..

[bib55] Holt C., Laury S. (2002). Risk aversion and incentive effects. Am. Econ. Rev..

[bib56] Iriberri N., Rey-Biel P. (2013). Elicited beliefs and social information in modified dictator games: what do dictator believe other dictators do?. Quant. Econ..

[bib57] Kerschbamer R. (2013). The Geometry of Distributional preferences and a Non-Parametric Identification Approach. Working Papers in Economics and Statistics 2013–25.

[bib58] Kirchsteiger G. (1994). The role of envy in ultimatum games. J. Econ. Behav. Organ..

[bib59] Konow J. (1993). Which is the fairest one of all? A positive analysis of justice theories. J. Econ. Lit..

[bib60] Kuziemko I., Buell R., Reich T., Norton M. (2014). “Last-Place Aversion”: evidence and redistributive implications. Q. J. Econ..

[bib61] Levine D. (1998). Modeling altruism and spitefulness in experiments. Rev. Econ. Dyn..

[bib62] Liebrand W. (1984). The effect of social motives, communication and group sizes on behavior in an n-person multi stage mixed motive game. Eur. J. Soc. Psychol..

[bib63] List J. (2007). On the interpretation of giving in dictator games. J. Polit. Econ..

[bib64] McFadden D., Zarembka P. (1974). Conditional logit analysis of qualitative choice behavior. Frontiers in Econometrics.

[bib65] Mui V. (1995). The economics of envy. J. Econ. Behav. Organ..

[bib66] Niederle M., Vesterlund L. (2007). Do women shy away from competition? Do men compete too much?. Q. J. Econ..

[bib67] Niederle M., Vesterlund L. (2010). Explaining the gender gap in math test scores: the role of competition. J. Econ. Perspect..

[bib68] Offerman T., Sonnemans J., Schram A. (1996). Value orientations, expectations and voluntary contributions in public goods. Econ. J..

[bib69] Rabin M. (1993). Incorporating fairness into game theory and economics. Am. Econ. Rev..

[bib71] Smith A. (1759). The Theory of Moral Sentiments.

[bib72] Sobel J. (2005). Interdependent preferences and reciprocity. J. Econ. Lit..

[bib73] Sonnemans J., Schram A., Offerman T. (1998). Public good provision and public bad prevention: the effect of framing. J. Econ. Behav. Organ..

[bib74] Sutter M., Haigner S., Kocher M. (2010). Choosing the stick or the carrot? – Endogenous institutional choice in social dilemma situations. Rev. Econ. Stud..

[bib76] Van Dijk F., Sonnemans J., van Winden F. (2002). Social ties in a public good experiment. J. Public Econ..

