# Supplementary material for: The geometry of distributional preferences and a non-parametric identification approach: The Equality Equivalence Test
Source: Eur Econ Rev. 2015 May;76:85–103. doi: 10.1016/j.euroecorev.2015.01.008 (PMC4459445; doi:10.1016/j.euroecorev.2015.01.008)
Supplement: Supplementary file 1 — Supplementary Material [file mmc1.doc]

**Online Appendix**

**for**

**The Geometry of Distributional Preferences**

**and a Non-Parametric Identification Approach:**

**The *Equality Equivalence Test***

Rudolf Kerschbamer

Department of Economics, University of Innsbruck[[1]](#footnote-2)#

This Version: January 2015

This appendix consists of two parts. Parts A discusses implementation issues for the case where the *Equality Equivalence Test* is used as a tool in experimental economics to answer specific research questions in which distributional preferences play an important role, to control for subject pool effects, or to help to interpret data from other (unrelated) experiments Appendix B contains the instructions to the experiments reported in Section 5 of the paper.

**Appendix A: Implementation Issues**

While the non-parametric identification approach proposed in the body of the paper seems in principle well suited as a tool in experimental economics to be added to arbitrary (other) experiments, there are several practical issues that need to be addressed.

**Role Assignment:** At least 3 different protocols regarding role assignment have been used in the literature on elicitation of distributional preferences, *fixed role assignment***,** where roles (active DM and passive person) are assigned ex ante, and only active DMs decide while passive persons do nothing (see, e.g., Cox, Sadiraj and Sadiraj 2008, and Cox and Sadiraj 2012); *role uncertainty***,** where each subject decides in the role of the active DM, and only later subjects get to know whether their decision is relevant – i.e., whether they have been chosen as DM or as passive person (this procedure was used by Engelmann and Strobel 2004 and by Blanco, Engelmann, and Normann 2011); and *double role assignment*, where each subject decides, and each subject gets two payoffs, one as an active DM and one as a passive person (as in Andreoni and Miller 2002, Anderoni and Vesterlund 2001 and in Fisman, Kariv, and Markovits 2007).[[2]](#footnote-3) While the fixed rule assignment (this protocol has been used to produce the data presented in body of the paper) seems to be the cleanest procedure from a theoretical point of view, it is not practicable when the test is intended as a tool to be added to arbitrary other experiments (since it would imply inviting twice as many subjects than needed for the main treatments). Each of the other two protocols seems to have some drawbacks. Consider the role uncertainty protocol first. Since it introduces an element of randomness in the determination of the payoff allocation resulting from a decision it raises theoretical questions related to the issue process fairness vs. outcome fairness (see Andreoni and Bernheim 2009 for evidence that some subjects care for process fairness). Secondly (and related to the first point), expectations about the behavior of the passive person in the counterfactual situation where she is the active DM might influence choices (provided process fairness matters; if not then not).[[3]](#footnote-4) The double rule assignment protocol seems to be better in the former dimension, but it might be worse in the latter as a subject's expectations about what she gets as a passive person and about what her passive person gets as the active DM are even more likely to enter the picture. In sum, both double rule assignment and role uncertainty protocol have their own problems and it is ultimately an empirical question which one performs better (in predicting the decisions in other distributional tasks, for instance). Here, promising evidence in support of the role uncertainty protocol is provided by Hedegaard et al. (2011). In their large-scale internet experiment they employ both the fixed role assignment and the role uncertainty protocol in a between subjects design and they show that the two protocols yield results that are statistically indistinguishable, both regarding the distribution of archetypes they yield and regarding the ability to predict behavior in other games.[[4]](#footnote-5)

**Presentation of the Binary Decision Problems:** In the paper-and-pen experiments reported in the body of the paper the binary decision tasks were presented to the subjects in *ordered lists* or tables (similar to the lists often used in risk-attitude elicitation tasks). In computer-aided experiments (using z-Tree developed by Fischbacher 2007, for instance) presenting the binary decisions *one-at-a-time in random order* (i.e., each binary decision on an own screen) might be an attractive alternative. Experience with both presentation techniques in computer-based experiments (where the test was added as a control at the end of the main experiments) suggests that the randomized test version produces (slightly) more inconsistencies (more than one switch in at least one of the two lists; or switches in the wrong direction) but might (slightly) increase the predictive power of the test for the classified subjects. This indicates that especially when the test is added as a control at the end of other experiments the presentation of the binary decision tasks might be critical in recovering reliable data on distributional preferences. The simplest procedure (presenting the binary decision tasks in a table) might not produce the most reliable results then, the main reason being that subjects do not think carefully enough about the tasks when they are presented all at once in a table.[[5]](#footnote-6) A "third way" to implement the test in the lab is to present the binary choices first in a totally randomized way (i.e., also randomized across blocks and in the presentation of the recurring alternative on the left or the right hand side), and to show subjects the ordered lists (with their decisions in the different choice tasks) when they are done with all choices. They can then revise their choices if they like.[[6]](#footnote-7) The latter procedure forces players to rethink their decisions; this might help to get fewer inconsistencies than in the random order design and might at the same time yield higher predictive power than the list versions. Again, it is ultimately an empirical question to sort out what the best way is to present the choice problems to the subjects when the test is used as a tool in experimental economics.

**Clustering of Subjects:** Clustering means dividing subjects into groups (clusters) so that members of one group are somehow similar to each other and dissimilar from members of other groups. There are many ways how clustering might be performed after having assigned to each subject an (*x, y*)-score. An obvious one is to cluster subjects into 9 groups corresponding to the 9 archetypes described in the body of the paper. This will often not meet the needs of the experimenter, though. The preferred way to group subjects will rather depend on at least three factors: (i) on the number of subjects taking part in the experiment; (ii) on the test version used; and (iii) on the research question under investigation.

*Number of Subjects:* If the number of subjects taking part in a study is small it does not make much sense to group them into too many clusters. Several approaches to divide subjects into 2-4 clusters spring to mind and the preferred one will, in general, depend on the other two dimensions discussed below. For instance, when a rough test version (high value of the quotient *s/q;* see the discussion in the next paragraph) is used a natural approach to divide subjects into 3-4 clusters is to use the sign of the two scores as the discriminator (both scores positive: altruistic; *x-*score negative, *y-*score positive: inequality averse; both scores negative: spiteful; *x-*score positive, *y*-score negative: equality averse; the latter class will be almost empty, though), while a finer test version would suggest that subjects with *x-* and *y-*score in {-0.5, +0.5} should be grouped in an own cluster (egoistic). On the other extreme, if the number of subjects taking part in the study is large there might be no need for exogenous clustering at all. For instance, in the experiments reported in Section 6 of the paper of the 36 points in the (*x, y*)-pane where subjects can potentially sit, more than half remained unoccupied, and only on nine points there was more than one subject sitting. Here it might make sense to work with nine clusters or less and to assign subjects who sit alone (or almost alone) at a point to one of the more frequented adjacent points according to some distance measure.

*Test Version*: One important design decision in the symmetric basic version of the test is the choice of the quotient *s/g* since this quotient determines the precision with which egoistic subjects are identified. If egoistic subjects are identified with high precision it might make sense to work with the following 5 clusters (where the last one is empty with high probability): *x*  {-0.5, +0.5} and *y*  {-0.5, +0.5}: egoistic; *x* ≥ 0.5 and *y* ≥ 0.5 and at least one inequality strict: altruistic (or efficiency loving); *x* ≤ 0.5 and *y* ≥ 0.5 and at least one inequality strict: inequality averse; *x* ≤ 0.5 and *y* ≤ 0.5 and at least one inequality strict: spiteful; and *x* ≥ 0.5 and *y* ≤ 0.5 and at least one inequality strict: equality averse.

*Research Question:* The research question under investigation is important for clustering, of course. For instance, for predicting the behavior of a subject in a standard dictator game, only the *y-*score should be important. So, for predicting behavior in dictator game like situations it might be sensible to divide subjects only according to this dimension (for instance, into 3 clusters, one with *y < 0,* the second with *y =* 0.5 and the third with *y >* 0.5; or, depending on the test design, into 4 or more clusters by splitting up the *y > 0.5* group in subgroups).

**Appendix B: Instructions (Translated from German)**

**Welcome and thank you for participating!**

You are taking part in an economic experiment on decision making. A research foundation has provided the funds for conducting the experiment. You can earn a considerable amount of money by participating. The text below will tell you how the amount you earn will be determined.

**Anonymity**

You will never be asked to reveal your identity to anyone during the decision-making part of the experiment. Neither the experimenters nor the other subjects will be able to link you to any of your decisions. In order to keep your decisions private, please do not reveal your choices to any other participant. The following means help to guarantee anonymity:

**Non-Computerized Experiment and Private Code**

The task you have to complete during the experiment is conducted in private on a printed form; that is, the experiment is not computerized. You have drawn a small sealed envelope from a box upon entering the room. PLEASE DO NOT OPEN YOUR ENVELOPE BEFORE THE EXPERIMENT STARTS. Your envelope contains your participation number. We will refer to it as "your private code" in the following. Your private code is the only identification used during the experiment and you will also need it to collect your cash payments.

When you have completed your task in the experiment you will be asked to write your private code on the front page of your form, to put the form in a new (larger) envelope, to seal the envelope, and to put it in a box located at the front door of the room you are sitting in. It is important that you do not write anything on the envelope, it should be left blank. It is also important that you keep the card with your private code: you need it to collect your earnings!

**Cash Payments**

Cash payments can be collected from tomorrow onwards in **room w.4.36** in the fourth floor (South/West) of this building. You will present your private code to an admin staff person (Mr. ...) and you will receive your cash payment in exchange. The admin staff person will not know who has done what and why, nor how payments were generated. No experimenter will be present in the room when you collect your money. Also, the private codes of this experiment will be mixed up with the codes of other experiments. This will again help to guarantee that the amount you earn cannot be linked to your decisions. Mr. ... is available from Monday to Friday between 9 a.m. and noon and between 2 p.m. and 3 p.m. in room w.4.36 in the fourth floor (South/West) of this building. Please collect your earnings within a weak. [You find those details also on the card displaying your private code.]

**Detailed Instructions**

**No Talking Allowed**

Please read this document carefully and do not talk to any other participant until the experiment is over. If there is anything that you don't understand, please raise your hand. An experimenter will approach you and clarify your questions in private. In about ten minutes this document (the front page included) will also be read aloud (by an experimenter).

**Two Groups and Two Different Tasks**

Before the experiment starts, the participants in this room will be randomly divided into two groups of equal size (see the text on the next page for details). The groups are called **Group A** and **Group B.** Members of Group A will be seated in this room, members of Group B will be seated in the adjacent laboratory. Each **member of Group A** will be asked to **make** a series of **ten decisions** that affect not only her or his own earnings but also the earnings of a member of Group B. The **members of Group B** **do not have a decision to make** in this experiment - their earnings will depend on the decisions of Group A members alone. Members of group B will be asked to fill out a questionnaire. This is their only task in this experiment.

**Matching**

After randomly assigning roles (member of Group A, or member of Group B) to participants, each member of Group A is anonymously paired with a member in Group B**.** The matching is 1:1; that is, each member of Group A is exactly matched with one member of Group B and vice versa. You will **never learn the identity of the member of the other group you are paired with.** In the same way, the member of the other group you are paired with will not learn your identity. In the following we call the member of the other group you are matched with **the other person.**

**Task of Members of Group A**

If you become a member of Group A you will be asked to make **ten decisions. In each of the ten decision problems** you are asked to decide between **two alternatives** which are called **LEFT** and **RIGHT.** Each alternative implies earnings for you and the other person. The ten decision problems will be presented as rows in a table. Note that only one of the ten decisions will be taken into consideration for the payoff determination - more on this below. Each decision problem will look like this:

| **LEFT** | | **Your Choice** | **RIGHT** | |
| --- | --- | --- | --- | --- |
| **you**  **receive** | **other person**  **receives** |  | **you**  **receive** | **other person**  **receives** |
| *a* Euros | *b* Euros | LEFT RIGHT | *c* Euros | *d* Euros |

The lower case letters in the cells of the decision problem displayed here are for illustration only, in the experiment the letters will be replaced by numbers. If you have been assigned the role of a member of Group A, if in this particular decision problem you choose LEFT, and if this particular problem is chosen as the payoff relevant one, then you receive earnings of *a* Euros while the other person will receives earnings of *b* Euros. Similarly, if you choose RIGHT, you receive *c* Euros and the other person receives *d* Euros. The table on the last page of this document displays the 10 decision problems each Group A member faces. The form members of Group A will receive will contain exactly two pages, the first page is an empty cover page, the second page contains the table on the last page of the current instructions (and nothing else)!

**Task of Members of Group B**

If you become a member of Group B you will be asked to fill out a two-page questionnaire. The form members of Group B will receive will contain exactly three pages, the first page is an empty cover page, the other two pages contain the questionnaire.

**Show-Up Fee**

Each participant in this experiment will receive a show-up fee of 4 Euros for participating. In addition, each participant receives earnings as specified in the next two paragraphs. That is, the **final payoff** of a participant **is the sum of** two parts, the **show-up fee plus** the **earnings in the experiment** (as specified below).

**Your Earnings if You Are a Member of Group A**

If you become a member of Group A your earnings and the earnings of the other person are determined as follows: At the end of the experiment (after you have made the ten choices in private), one of the 10 decision problems will be randomly selected as the payoff-relevant one. For this purpose an experimenter with a bingo cage containing ten balls numbered 1-10 will go from one member of Group A to the next starting on the leftmost cubicle of the first row. Please make sure that your completed form is closed when the experimenter approaches you. The experimenter will ask you to draw one of the balls with the device designated for that purpose. **The number on the ball gives the decision task that will be used to determine your earning and that of the other person.** Your actual earnings and those of the other person correspond exactly to the payoffs in the alternative (LEFT or RIGHT) you have chosen in that specific decision problem. You will be asked by the experimenter to write the number of the payoff-relevant decision problem on the cover page of your form. You (but no one else) will then be given the opportunity to take in private a look at your choice in the payoff-relevant decision problem. Then you will be asked to label (in private) the cover sheet of the form with your private code and to seal the form in the envelope.

**Your Earnings if You Are a Member of Group B**

In addition to the 4 Euros show-up fee each member of Group B will receive the earnings as described in the previous paragraph from exactly one member of Group A.

**Role Assignment and Start of the Experiment**

After the instructions at hand have been red aloud and all questions have been answered you (and all other participants in this room) will be asked to open the sealed envelope you draw from the box when entering this room. The envelope contains a card with your private code. The code ends with a number. If this number is even, you are a member of Group A, if it is odd, you are a member of Group B. Members of Group A are asked to take a seat at one of the computer terminals with sliding walls in this room. Members of Group B will be escorted to the adjacent room and asked to take a seat at one of the computer terminals with sliding walls in that room. In both rooms computers are (and will remain) switched off. An experimenter will then distribute the forms in each room. Members of Group A will receive a form that contains an empty cover page and a page containing the decision tasks displayed on the next page, members of Group B will receive a form that contains an empty cover page and a two-page questionnaire.

**The End of the Experiment**

After you have completed your task you will be asked to write your private code on the empty cover page of your form. PLEASE WAIT UNTIL YOU ARE ASKED BEFORE WRITING THE CODE ON THE COVER. Then put the form in the envelope and seal it. Upon leaving the room you are asked to put the envelope in the box located near the front door of the room you are sitting in.

**The Ten Decision Tasks for Members of Group A**

The table below displays the ten decision problems presented to members of Group A. Members of Group A will be asked to mark in each row whether they prefer the alternative on the left hand side (LEFT) or the alternative on the right hand side (RIGHT). They have to decide for ONE of the two alternatives in each of the ten rows.

**The table below is for illustration only. After the role assignment, members of Group A will receive a form that contains two pages, an empty cover page and a page containing exactly the table below (and nothing else).**

| **Dec. Nr.** | **LEFT** | | **Your Choice** | **RIGHT** | |
| --- | --- | --- | --- | --- | --- |
|  | **you**  **receive** | **other person**  **receives** |  | **you**  **receive** | **other person**  **receives** |
| 1 | **8 Euros** | **13 Euros** | LEFT RIGHT | **10 Euros** | **10 Euros** |
| 2 | **9 Euros** | **13 Euros** | LEFT RIGHT | **10 Euros** | **10 Euros** |
| 3 | **10 Euros** | **13 Euros** | LEFT RIGHT | **10 Euros** | **10 Euros** |
| 4 | **11 Euros** | **13 Euros** | LEFT RIGHT | **10 Euros** | **10 Euros** |
| 5 | **12 Euros** | **13 Euros** | LEFT RIGHT | **10 Euros** | **10 Euros** |

| **Dec. Nr.** | **LEFT** | | **Your Choice** | **RIGHT** | |
| --- | --- | --- | --- | --- | --- |
|  | **you**  **receive** | **other person**  **receives** |  | **you**  **receive** | **other person**  **receives** |
| 6 | **8 Euros** | **7 Euros** | LEFT RIGHT | **10 Euros** | **10 Euros** |
| 7 | **9 Euros** | **7 Euros** | LEFT RIGHT | **10 Euros** | **10 Euros** |
| 8 | **10 Euros** | **7 Euros** | LEFT RIGHT | **10 Euros** | **10 Euros** |
| 9 | **11 Euros** | **7 Euros** | LEFT RIGHT | **10 Euros** | **10 Euros** |
| 10 | **12 Euros** | **7 Euros** | LEFT RIGHT | **10 Euros** | **10 Euros** |

1. ^#^ Universitätsstrasse 15, A-6020 Innsbruck, Austria; e-mail: Rudolf.Kerschbamer@uibk.ac.at; phone: ++43 512 507 7400 [↑](#footnote-ref-2)
2. Actually, the double role assignment protocol comes in two varieties; while in version 1 (the version discussed in the main text) the computer program makes sure that a subject's active DM is a different participant than a subject's passive person (and instructions are very explicit about this), version 2 (often used in the implementation of the ring-test and in related tests designed by social psychologists) has fixed pairs, meaning that a subject's active DM is the same participant as her passive person. Version 2 seems theoretically problematic and is therefore ignored in the discussion in the main text. [↑](#footnote-ref-3)
3. If subjects have distributional preferences in the textbook variety (only outcomes matter) then expectations should not shape decisions in the role uncertainty protocol: with some probability α (= ½) the other person is the active DM und your expectations about her/his behavior influence what you expect to get in that case; given your preferences and expectations this yields a fixed "utility" that you get with probability α; to maximize your overall (expected) utility you still have an incentive to maximize that part of your "utility" that realizes with probability 1 - α. No expectations enter in that part of your overall expected utility. The story changes if (some) subjects are not (only) concerned with the "fairness" of outcomes but (also) with the "fairness" of lotteries. This is beyond pure distributional preferences, though. [↑](#footnote-ref-4)
4. This is in line with earlier evidence provided by Engelmann and Strobel (2004) who find relatively small and insignificant differences in the choices of subjects between their main treatment with role uncertainty and their control with fixed role assignment. [↑](#footnote-ref-5)
5. Misclassification of subjects who do not think carefully enough about the alternatives in a binary decision task seems less of an issue in the experiments reported in the body of the paper because subjects' task was merely to make 10 binary decisions there. We therefore opted for a design that is cleaner in terms of experimenter demand and audience effects. [↑](#footnote-ref-6)
6. Hedegaard et al. (2011) employ this protocol in their internet experiments. [↑](#footnote-ref-7)
