# Supplementary material for: The geometry of distributional preferences and a non-parametric identification approach: The Equality Equivalence Test
Source: Eur Econ Rev. 2015 May;76:85–103. doi: 10.1016/j.euroecorev.2015.01.008 (PMC4459445; doi:10.1016/j.euroecorev.2015.01.008)
Supplement: Supplementary file 2 — Supplementary Material [file mmc2.doc]

**Replication Data**

**for**

**The Geometry of Distributional Preferences**

**and a Non-Parametric Identification Approach:**

**The *Equality Equivalence Test***

Rudolf Kerschbamer

Department of Economics, University of Innsbruck[[1]](#footnote-2)#

This Version: January 2015

Section 5 of the paper reports the results of a paper-and-pen experiment based on the symmetric basic version of the *Equality Equivalence Test.* The experimental procedures are detailed in Section 5 of the manuscript. Part A of this file (identical to Part B of the Online Appendix) contains the instructions to the experiment. In the experiment subjects were asked to make ten binary decisions. The ten decision tasks are displayed in the table on page 5 of this document. Part B of this file (based on Subsection 4.4 of the manuscript) explains how the data patterns produced by the subjects are translated into a two-dimensional index of type and intensity of distributional concerns. Part C of the file (same contents as Figure 6 in the paper) contains the data points collected in the experiment.

**Part A: Instructions (Translated from German)**

**Welcome and thank you for participating!**

You are taking part in an economic experiment on decision making. A research foundation has provided the funds for conducting the experiment. You can earn a considerable amount of money by participating. The text below will tell you how the amount you earn will be determined.

**Anonymity**

You will never be asked to reveal your identity to anyone during the decision-making part of the experiment. Neither the experimenters nor the other subjects will be able to link you to any of your decisions. In order to keep your decisions private, please do not reveal your choices to any other participant. The following means help to guarantee anonymity:

**Non-Computerized Experiment and Private Code**

The task you have to complete during the experiment is conducted in private on a printed form; that is, the experiment is not computerized. You have drawn a small sealed envelope from a box upon entering the room. PLEASE DO NOT OPEN YOUR ENVELOPE BEFORE THE EXPERIMENT STARTS. Your envelope contains your participation number. We will refer to it as "your private code" in the following. Your private code is the only identification used during the experiment and you will also need it to collect your cash payments.

When you have completed your task in the experiment you will be asked to write your private code on the front page of your form, to put the form in a new (larger) envelope, to seal the envelope, and to put it in a box located at the front door of the room you are sitting in. It is important that you do not write anything on the envelope, it should be left blank. It is also important that you keep the card with your private code: you need it to collect your earnings!

**Cash Payments**

Cash payments can be collected from tomorrow onwards in **room w.4.36** in the fourth floor (South/West) of this building. You will present your private code to an admin staff person (Mr. ...) and you will receive your cash payment in exchange. The admin staff person will not know who has done what and why, nor how payments were generated. No experimenter will be present in the room when you collect your money. Also, the private codes of this experiment will be mixed up with the codes of other experiments. This will again help to guarantee that the amount you earn cannot be linked to your decisions. Mr. ... is available from Monday to Friday between 9 a.m. and noon and between 2 p.m. and 3 p.m. in room w.4.36 in the fourth floor (South/West) of this building. Please collect your earnings within a weak. [You find those details also on the card displaying your private code.]

**Detailed Instructions**

**No Talking Allowed**

Please read this document carefully and do not talk to any other participant until the experiment is over. If there is anything that you don't understand, please raise your hand. An experimenter will approach you and clarify your questions in private. In about ten minutes this document (the front page included) will also be read aloud (by an experimenter).

**Two Groups and Two Different Tasks**

Before the experiment starts, the participants in this room will be randomly divided into two groups of equal size (see the text on the next page for details). The groups are called **Group A** and **Group B.** Members of Group A will be seated in this room, members of Group B will be seated in the adjacent laboratory. Each **member of Group A** will be asked to **make** a series of **ten decisions** that affect not only her or his own earnings but also the earnings of a member of Group B. The **members of Group B** **do not have a decision to make** in this experiment - their earnings will depend on the decisions of Group A members alone. Members of group B will be asked to fill out a questionnaire. This is their only task in this experiment.

**Matching**

After randomly assigning roles (member of Group A, or member of Group B) to participants, each member of Group A is anonymously paired with a member in Group B**.** The matching is 1:1; that is, each member of Group A is exactly matched with one member of Group B and vice versa. You will **never learn the identity of the member of the other group you are paired with.** In the same way, the member of the other group you are paired with will not learn your identity. In the following we call the member of the other group you are matched with **the other person.**

**Task of Members of Group A**

If you become a member of Group A you will be asked to make **ten decisions. In each of the ten decision problems** you are asked to decide between **two alternatives** which are called **LEFT** and **RIGHT.** Each alternative implies earnings for you and the other person. The ten decision problems will be presented as rows in a table. Note that only one of the ten decisions will be taken into consideration for the payoff determination - more on this below. Each decision problem will look like this:

| **LEFT** | | **Your Choice** | **RIGHT** | |
| --- | --- | --- | --- | --- |
| **you**  **receive** | **other person**  **receives** |  | **you**  **receive** | **other person**  **receives** |
| *a* Euros | *b* Euros | LEFT RIGHT | *c* Euros | *d* Euros |

The lower case letters in the cells of the decision problem displayed here are for illustration only, in the experiment the letters will be replaced by numbers. If you have been assigned the role of a member of Group A, if in this particular decision problem you choose LEFT, and if this particular problem is chosen as the payoff relevant one, then you receive earnings of *a* Euros while the other person will receives earnings of *b* Euros. Similarly, if you choose RIGHT, you receive *c* Euros and the other person receives *d* Euros. The table on the last page of this document displays the 10 decision problems each Group A member faces. The form members of Group A will receive will contain exactly two pages, the first page is an empty cover page, the second page contains the table on the last page of the current instructions (and nothing else)!

**Task of Members of Group B**

If you become a member of Group B you will be asked to fill out a two-page questionnaire. The form members of Group B will receive will contain exactly three pages, the first page is an empty cover page, the other two pages contain the questionnaire.

**Show-Up Fee**

Each participant in this experiment will receive a show-up fee of 4 Euros for participating. In addition, each participant receives earnings as specified in the next two paragraphs. That is, the **final payoff** of a participant **is the sum of** two parts, the **show-up fee plus** the **earnings in the experiment** (as specified below).

**Your Earnings if You Are a Member of Group A**

If you become a member of Group A your earnings and the earnings of the other person are determined as follows: At the end of the experiment (after you have made the ten choices in private), one of the 10 decision problems will be randomly selected as the payoff-relevant one. For this purpose an experimenter with a bingo cage containing ten balls numbered 1-10 will go from one member of Group A to the next starting on the leftmost cubicle of the first row. Please make sure that your completed form is closed when the experimenter approaches you. The experimenter will ask you to draw one of the balls with the device designated for that purpose. **The number on the ball gives the decision task that will be used to determine your earning and that of the other person.** Your actual earnings and those of the other person correspond exactly to the payoffs in the alternative (LEFT or RIGHT) you have chosen in that specific decision problem. You will be asked by the experimenter to write the number of the payoff-relevant decision problem on the cover page of your form. You (but no one else) will then be given the opportunity to take in private a look at your choice in the payoff-relevant decision problem. Then you will be asked to label (in private) the cover sheet of the form with your private code and to seal the form in the envelope.

**Your Earnings if You Are a Member of Group B**

In addition to the 4 Euros show-up fee each member of Group B will receive the earnings as described in the previous paragraph from exactly one member of Group A.

**Role Assignment and Start of the Experiment**

After the instructions at hand have been red aloud and all questions have been answered you (and all other participants in this room) will be asked to open the sealed envelope you draw from the box when entering this room. The envelope contains a card with your private code. The code ends with a number. If this number is even, you are a member of Group A, if it is odd, you are a member of Group B. Members of Group A are asked to take a seat at one of the computer terminals with sliding walls in this room. Members of Group B will be escorted to the adjacent room and asked to take a seat at one of the computer terminals with sliding walls in that room. In both rooms computers are (and will remain) switched off. An experimenter will then distribute the forms in each room. Members of Group A will receive a form that contains an empty cover page and a page containing the decision tasks displayed on the next page, members of Group B will receive a form that contains an empty cover page and a two-page questionnaire.

**The End of the Experiment**

After you have completed your task you will be asked to write your private code on the empty cover page of your form. PLEASE WAIT UNTIL YOU ARE ASKED BEFORE WRITING THE CODE ON THE COVER. Then put the form in the envelope and seal it. Upon leaving the room you are asked to put the envelope in the box located near the front door of the room you are sitting in.

**The Ten Decision Tasks for Members of Group A**

The table below displays the ten decision problems presented to members of Group A. Members of Group A will be asked to mark in each row whether they prefer the alternative on the left hand side (LEFT) or the alternative on the right hand side (RIGHT). They have to decide for ONE of the two alternatives in each of the ten rows.

**The table below is for illustration only. After the role assignment, members of Group A will receive a form that contains two pages, an empty cover page and a page containing exactly the table below (and nothing else).**

| **Dec. Nr.** | **LEFT** | | **Your Choice** | **RIGHT** | |
| --- | --- | --- | --- | --- | --- |
|  | **you**  **receive** | **other person**  **receives** |  | **you**  **receive** | **other person**  **receives** |
| 1 | **8 Euros** | **13 Euros** | LEFT RIGHT | **10 Euros** | **10 Euros** |
| 2 | **9 Euros** | **13 Euros** | LEFT RIGHT | **10 Euros** | **10 Euros** |
| 3 | **10 Euros** | **13 Euros** | LEFT RIGHT | **10 Euros** | **10 Euros** |
| 4 | **11 Euros** | **13 Euros** | LEFT RIGHT | **10 Euros** | **10 Euros** |
| 5 | **12 Euros** | **13 Euros** | LEFT RIGHT | **10 Euros** | **10 Euros** |

| **Dec. Nr.** | **LEFT** | | **Your Choice** | **RIGHT** | |
| --- | --- | --- | --- | --- | --- |
|  | **you**  **receive** | **other person**  **receives** |  | **you**  **receive** | **other person**  **receives** |
| 6 | **8 Euros** | **7 Euros** | LEFT RIGHT | **10 Euros** | **10 Euros** |
| 7 | **9 Euros** | **7 Euros** | LEFT RIGHT | **10 Euros** | **10 Euros** |
| 8 | **10 Euros** | **7 Euros** | LEFT RIGHT | **10 Euros** | **10 Euros** |
| 9 | **11 Euros** | **7 Euros** | LEFT RIGHT | **10 Euros** | **10 Euros** |
| 10 | **12 Euros** | **7 Euros** | LEFT RIGHT | **10 Euros** | **10 Euros** |

Part B: Identifying Archetype and Characterizing Intensity of Distributional Concerns

Determination of (*x, y*)-Score

| subject chooses Left for the 1^st^ time in row | in the *X*-list (Dec. Nr. 1-5)  *x*-score | in the *Y*-list (Dec. Nr. 5-10)  *y*-score |
| --- | --- | --- |
| 1 | 2.5 | - 2.5 |
| 2 | *1*.5 | - 1.5 |
| 3 | 0.5 | - 0.5 |
| 4 | - 0.5 | 0.5 |
| 5 | - 1.5 | 1.5 |
| never | - 2.5 | 2.5 |

**Part C: Observed Data Points**

|  | | **x-score** | | | | | |
| --- | --- | --- | --- | --- | --- | --- | --- |
|  |  | **- 2.5** | **-1.5** | **-0.5** | **+0.5** | **+1.5** | **+2.5** |
| **y-score** | **-2.5** | 1 | 0 | 0 | 0 | 1 | 0 |
|  | **-1.5** | 0 | 0 | 0 | 0 | 0 | 0 |
|  | **-0.5** | 1 | 1 | 3 | 2 | 0 | 0 |
|  | **+0.5** | 1 | 1 | 11 | 29 | 0 | 1 |
|  | **+1.5** | 0 | 0 | 2 | 3 | 2 | 1 |
|  | **+2.5** | 22 | 0 | 0 | 15 | 10 | 0 |

1. ^#^ Universitätsstrasse 15, A-6020 Innsbruck, Austria; e-mail: Rudolf.Kerschbamer@uibk.ac.at; phone: ++43 512 507 7400 [↑](#footnote-ref-2)
